# CircRNA DICAR as a novel endogenous regulator for diabetic cardiomyopathy and diabetic pyroptosis of cardiomyocytes

**DOI:** 10.1038/s41392-022-01306-2

**Published:** 2023-03-08

**Authors:** Qiong Yuan, Yunwei Sun, Fan Yang, Dan Yan, Meihua Shen, Zhigang Jin, Lin Zhan, Guangqi Liu, Ling Yang, Qianyi Zhou, Zhijun Yu, Xiangyu Zhou, Yang Yu, Yong Xu, Qingming Wu, Jianfang Luo, Xiamin Hu, Chunxiang Zhang

**Affiliations:** 1 Department of Cardiology, the Affiliated Hospital of Southwest Medical University and Key Laboratory of Medical Electrophysiology, Ministry of Education, Institute of Cardiovascular Research and Institute of Metabolic Diseases, Southwest Medical University, Luzhou, 646000 China; 2 College of Medicine, Wuhan University of Science and Technology, Wuhan, 430065 China; 3 Department of Emergency and Critical Care Medicine, Guangdong Provincial People’s Hospital, Guangdong Academy of Medical Sciences, Guangzhou, 510080 China; 4 China Resource & WISCO General Hospital, Wuhan University of Science and Technology, Wuhan, 430065 China; 5 Department of Cardiology, Guangdong Cardiovascular Institute, Guangdong Provincial Key Laboratory of Coronary Heart Disease Prevention, Guangdong General Hospital, Guangzhou, China; 6 College of Pharmacy, Shanghai University of Medicine and Health Sciences, Shanghai, 210000 China

**Keywords:** Non-coding RNAs, Cardiovascular diseases, Molecular medicine

## Abstract

In this study, we identified that a conserved circular RNA (circRNA) DICAR, which was downregulated in diabetic mouse hearts. DICAR had an inhibitory effect on diabetic cardiomyopathy (DCM), as the spontaneous cardiac dysfunction, cardiac cell hypertrophy, and cardiac fibrosis occurred in DICAR deficiency (*DICAR*^*+/−*^) mice, whereas the DCM was alleviated in DICAR-overexpressed *DICAR*^*Tg*^ mice. At the cellular level, we found that overexpression of DICAR inhibited, but knockdown of DICAR enhanced the diabetic cardiomyocyte pyroptosis. At the molecular level, we identified that DICAR-VCP-Med12 degradation could be the underlying molecular mechanism in DICAR-mediated effects. The synthesized DICAR junction part (DICAR-JP) exhibited a similar effect to the entire DICAR. In addition, the expression of DICAR in circulating blood cells and plasma from diabetic patients was lower than that from health controls, which was consistent with the decreased DICAR expression in diabetic hearts. DICAR and the synthesized DICAR-JP may be drug candidates for DCM.

## Introduction

Circular RNA (circRNA) belongs to the non-coding RNA (ncRNA) group, which is ubiquitous, stable, and evolutionarily conserved among the eukaryotes.^1^ Structurally speaking, circRNAs are covalently closed at the 5’ to 3’ ends, which are more resistant to exonucleases than linear RNAs.^2,3^ In addition, a circRNA is able to form the loop structure, which is more stable structure in RNA. Indeed, it has been reported that the half-life of circRNA is about 24.56 ± 5.2 h, which is significantly longer than mRNA (**≈**16.4 h).^4^ The unique structure of circRNAs suggests that circRNAs may play more stable effects within the cells.

Diabetic cardiomyopathy (DCM) is defined as the abnormal myocardial structure and performance that is induced by diabetes, but is not caused by its underlying hypertension, coronary artery disease or valvular disease.^5^ DCM is characterized by adverse structural remodeling (including cardiac hypertrophy and fibrosis), early-onset diastolic dysfunction, and late-onset systolic dysfunction.^6^ Clearly, diabetic cardiomyopathy is a metabolic heart disease, in which hyperglycemia and related metabolic and endocrine disorders are the triggering factors for the myocardial damages through multiple mechanisms including energy metabolism imbalance described in our previous study.^7^ Due to the limited knowledge regarding the detailed molecular mechanisms responsible for the pathogenesis of diabetic cardiomyopathy, there are still lack of any specific molecular treatments for this frequently encountered heart disease.

Several kinds of programmed cell death are reported to be involved in the development of diabetic cardiomyopathy, among them apoptosis are well-studied.^8,9^ Pyroptosis is a newly identified form of programmed cell death, which could induce cell lysis and the release of pro-inflammatory factors (such as IL-1β and IL-18) and high-mobility group box-1 protein (HMGB1).^10^ Recently, Shao et al.^11^ defined pyroptosis as gasdermin-induced necrotic cell death and applied this term to all gasdermin family members-induced cell death through membrane permeabilization. It is established that the cardiomyocyte pyroptosis plays an important role in the development of DCM.^1^ Silencing NLRP3 was reported to suppress pyroptosis in H9c2 cardiomyocytes under high-glucose concentration and improve cardiac inflammation, pyroptosis, and fibrosis in Type 2 diabetes mellitus (T2DM) rats.^12^ In addition, caspase-1-induced pyroptosis in DCM is related to ncRNAs including microRNA-30 (miR-30).^13^ and long ncRNA (lncRNA) Kcnq1ot1c.^14^ Therefore, we believe that inhibiting of cell pyroptosis may offer a new therapeutic strategy for diabetic cardiomyopathy.^10^

It has been reported that circRNAs extensively participate in the development of various diseases, including cancer, cardiovascular diseases and T2DM.^15,16^ However, only a few studies on circRNAs are found to be associated with the DCM. In this respect, Yang et al.^17^ reported that hsa_circ_0076631 could regulate the caspase-1-inducd pyorptosis by targeting miR-214-3p in DCM. CircularRNA circ_0071269 knockdown is found to protect against from DCM by the microRNA-145/gasdermin A axis.^18^ In addition, the increased expression of circRNA_000203 in mouse diabetic hearts could enhance the expression of fibrosis-associated genes by sponging miR-26b-5p.^19^ A circRNA cerebellar degeneration-related protein 1 antisense (CDR1as) is found to be upregulated in DCM. CDR1as activates the Hippo signaling pathway by inhibiting of the mammalian sterile 20-like kinase 1 (MST1) ubiquitination level, which could induce the apoptosis in cardiomyopathy.^20^ Another circRNA circHIPK3 may downregulate PTEN to protect cardiomyocytes from high glucose-induced cell apoptosis.

In this study, we have identified that circRNA mm9_circ_008009 is downregulated in mouse hearts with DCM and in cardiomyocytes treated with the advanced glycation end products (AGEs). We named it as well as its conserved human circular RNA hsa_circ_0131202 as DICAR (the **d**iabetes-**i**nduced **ci**rculation-**a**ssociated circular **R**NA). DICAR could efficiently inhibit the pyroptosis via binding with valosin-containing protein (VCP) and blocking of the Med12 protein degradation. Synthetic DICAR-junction part (DICAR-JP) could rescue the cardiomyocytes pyroptosis induced by AGEs, suggesting its potential as a therapeutic candidate for diabetes–associated cardiac impairments.

## Results

### DICAR is associated with diabetic cardiomyopathy in mice

**Fig. 1:**
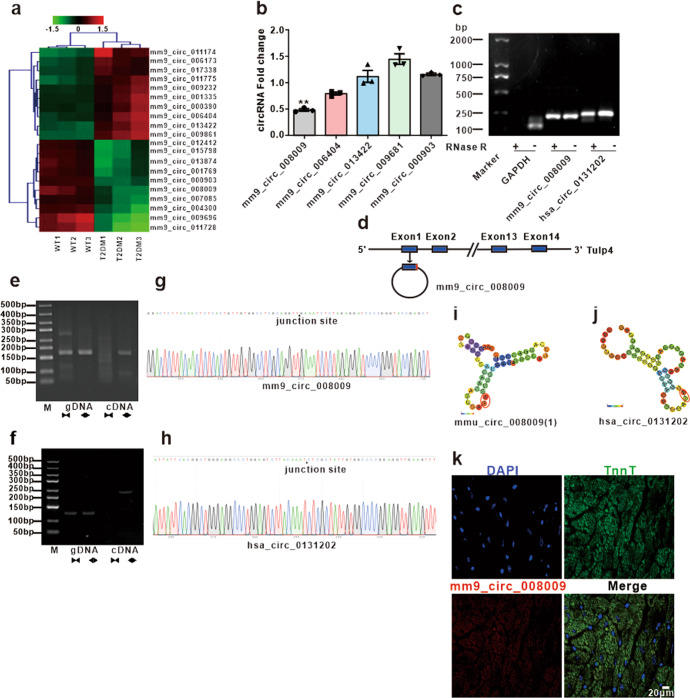
Identified circRNA expression in diabetic cardiomyopathy mouse. **a** Microarray identified in the hearts of db/db mice. **b** Select significantly regulated circRNA detected by qPCR (*n* = 9). **c** CircRNA resistance detected by RNAse. **d** Mm9-circ-008009 structure. **e** Identification of the mm9-circ-008009 back-spliced junction sites. **f** Identification of the hsa-circ-0131202 back-spliced junction sites. **g**, **h** Sanger sequence of mm9-circ-008009 and hsa-circ-0131202. **i**, **j** Second structure prediction of mm9-circ-008009 and hsa-circ-0131202. **k** FISH detection of mm9-circ-008009 in the mouse heart tissues. Data are represented as mean ± SEM. ^**^P < 0.01 *vs*. WT group

We screened the circRNA expression profiles in heart tissues from wild-types (WT) mice and diabetic db/db mice by circRNA microarray. The results revealed that 58 circRNAs with high homology between human and mouse were differentially expressed between the two groups by at least 1.5-fold (*P* < 0.05) (GSE199133). The representative results of circRNA microarray from two groups were shown in Fig. 1a. We next selected five circRNAs with the highest fold change values for further validation by RT-qPCR (Fig. 1b). We found that mm9_circ_008009 was the most decreased circRNA in the diabetic hearts (Fig. 1b). The mm9_circ_008009 was resistant to ribonuclease R digestion, whereas linear GAPDH mRNA was found to be easily degraded (Fig. 1c). According to the circBase description, the parent gene of mm9_circ_008009 is Tulp4. We found that mm9_circ_008009 was formed from the exon1 and intron of Tulp4 (Fig. 1d). The divergent primers were designed to amplify mm9_circ_008009, while the convergent primers were designed to amplify the corresponding linear mRNA using cDNA and genomic DNA (gDNA) in the mouse cardiomyocytes (MCMs). Both mm9_circ_008009 and hsa_circ_0131202 were amplified by divergent primers in cDNA, but not in gDNA, respectively (Fig. 1e, f), indicating that the circRNA species are circular in form. Sanger sequencing analysis of the PCR products demonstrated that mm9_circ_008009 was generated from exon 1 and intron of *Tulp4* through the “back splices” mechanism (Fig. 1g). We blasted the sequences between mm9_circ_008009 and 36 human circRNAs transcripted from *TULP4* based on circBase and found that the conserved identity between hsa_circ_0131202 and mm9_circ_008009 was 79.65%. In addition, the circRNA characteristic of hsa_circ_0131202 was identified, and the RNA was detected with divergent primers in cDNA, but not in gDNA of human cardiomyocytes (HCMs) (Fig. 1f). Sequence analyses revealed that approximately 20-bp fragment of the mm9_circ_008009 junction site (Fig. 1g) was similar with hsa_circ_0131202 (Fig. 1h). Moreover, the RNA models structure of hsa_circ_0131202 (Fig. 1j), which includes the back-splicing junction (BSJ), was similar to that of mm9_circ_008009 (Fig. 1i). We also predicted other human circRNAs from TULP4 (Supplementary Fig. S1); however, the deduced structures were not similar to those of mm9_circ_008009. Therefore, hsa_circ_013202 and mm9_circ_008009 with the same source, and similar structure and sequence may have the same or similar molecular and biological functions. The fluorescence in situ hybridization (FISH) experiment demonstrated that mm9_circ_008009 was mainly located in the cytoplasm of cardiac tissue cells (Fig. 1k), cultured MCMs and HCMs (Supplementary Fig. S2a). Advanced glycation end-products (AGEs) are believed to be involved in diverse complications of diabetes. The expression of mm9_circ_008009 and hsa_circ_0131202 in MCMs and HCMs was downregulated when treated by AGEs in a dose-dependent manner (Supplementary Fig. S2b, c). Therefore, we named mm9_circ_008009 and hsa_circ_0131202 as the diabetes-induced circulation-associated circRNAs (DICAR).

### Cardiac dysfunction, cardiac cell hypertrophy, and cardiac fibrosis are found in *DICAR*^*+/−*^ mice

To test the potential role of DICAR in diabetic cardiomyopathy, we created the DICAR knock-out mice. Unfortunately, we did not obtain the homozygous DICAR knockout mice (*DICAR*^*−/−*^ mice), which might be possibly lethal to mice. In heterozygous DICAR knockout (*DICAR*^*+/−*^) mice, the knockdown efficiency and specificity of DICAR were verified by qRT-PCR. As illustrated in supplementary Fig. S3a, DICAR was expressed at a very low level, while Tulp4 mRNA was not affected in the *DICAR*^*+/−*^ mice. The results revealed that the DICAR knockdown did not affect the expression of the parent gene *Tulp4* (Supplementary Fig. S3a).

**Fig. 2:**
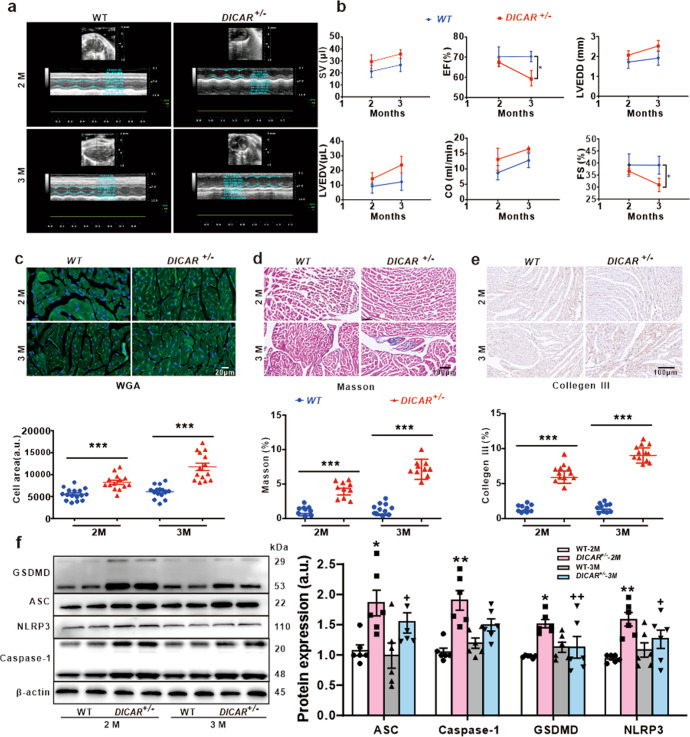
Effect of DICAR on the cardiac functions and remodeling. **a** Echocardiography images of *DICAR*^*+/−*^ mouse. **b** Summary of the heart functions, including stroke volume (SV), ejection fraction (EF), left ventricular end-diastolic dimension (LVEDD), cardiac output (CO), fractional shortening (FS). **c** Representative images and summary data of wheat germ agglutinin (WGA) staining. **d** Representative images and summary data of Masson staining of *DICAR*^*+/−*^. **e** Representative IHF images and summary data of collagen III. **f** Representative and summary data of WB images of pyroptosis detected in the heart tissues. Data are represented as mean ± SEM. ^*^P < 0.05, ^**^P < 0.01 vs. WT group, *n* = 6–8

Interestingly, we found that the cardiac function was impaired in heterozygous DICAR-deficient (*DICAR*^*+/−*^) mice compared with their control mice. The results of echocardiographic assessment demonstrated that, when compared with wild type (WT) control mice at same age, the increased left ventricular end-systolic volume (LVESV), left ventricular end-diastolic volume (LVEDV), and left ventricular end-diastolic dimension (LVEDD) were noted in *DICAR*^*+/−*^ mice (Fig. 2a, b). At 3-month-old, the decreased ejection fraction (EF) and fractional shortening (FS) were noted in *DICAR*^*+/−*^ mice (Fig. 2a, b). The results demonstrated that the decreased expression of DICAR impaired the cardiac function, which is similar to the cardiac dysfunction in diabetic patients.^21^ To test the potential role of DICAR in cardiac remodeling and fibrosis, we analyzed the area of cardiomyocytes and fibrosis in *DICAR*^*+/−*^ mice. When compared with these in WT mice, age-matched *DICAR*^*+/*−^ mice demonstrated a marked increase in the cardiomyocyte size determined by the wheat germ agglutinin (WGA) staining (Fig. 2c), a marked increase in collagen deposition in the myocardial interstitium determined by Masson staining (Fig. 2d), and the enhanced expression of collagen III (Fig. 2e), which reflected the cardiac remodeling.^22^ These data suggested that the deficiency of DICAR in *DICAR*^*+/−*^ mice could induce cardiac dysfunction, hypertrophy, and fibrosis in vivo.

Cardiac cell pyroptosis is a key cellular event in diabetic hearts. To test the effect of DICAR on pyroptosis, we detected a set of pyroptosis-related biomarkers in the heart tissues of *DICAR*^*+/−*^ mice. In this experiment, the expression of cleaved GSDMD, ASC, NLRP3, and caspase-1 p21 proteins were used to evaluate the activation of pyroptosis. The results revealed that all these protein markers of pyroptosis were upregulated in the heart tissues from *DICAR*^*+/−*^ mice compared to those in WT controls (Fig. 2f).

### *DICAR*^*Tg*^ mice are resistant to heart damages induced by diabetes

**Fig. 3:**
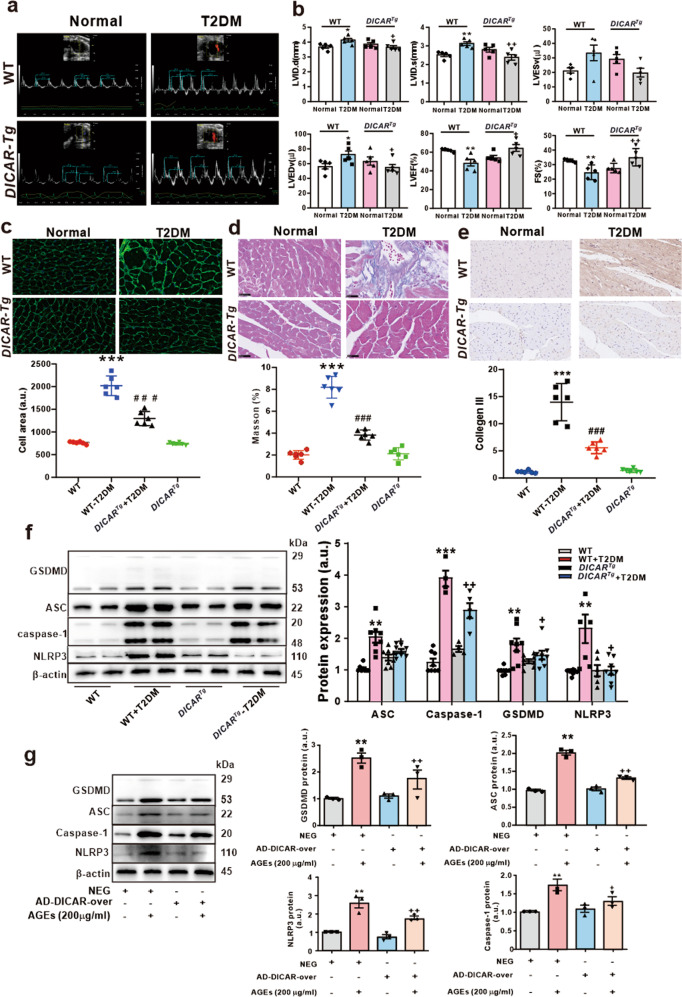
Gain of DICAR function ameliorates T2DM-induced heart function in *DICAR*^*Tg*^ mouse. **a** Representative echocardiography images of *DICAR*^*Tg*^ mouse. **b** The DICAR overexpression ameliorates heart functions, including stroke volume (SV), ejection fraction (EF), left ventricular end-diastolic dimension (LVEDD), left ventricular end-diastolic volume (LVEDV), cardiac output (CO), and fractional shortening (FS). **c** The DICAR overexpression decreased the cardiomyocytes area detected by wheat germ agglutinin (WGA) staining. **d** Collagen deposition detected by Masson staining. **e** Collagen III expression detected by IHC. **f** In the *DICAR*^*Tg*^ mouse, the DICAR overexpression inhibited the pyroptosis of heart tissue induced by T2DM that detected by the GSDMD, ASC, IL-1β, and NLRP3 protein expression. Data are represented as mean ± SEM. ^**^P < 0.01 vs. Normal, ^++^*P* < 0.01 vs. Normal + T2DM, *n* = 8. **g** Adenovirus (AD)-DICAR infected HL-1 cells inhibited the pyroptosis induced by AGEs (200 µg/mL). Data are represented as mean ± SEM. ^**^P < 0.01 *vs*. NEG, ^++^*P* < 0.01 vs. AD-DICAR-over, *n* = 4

To further investigate the effect of DICAR on the development of DCM, *DICAR*^*Tg*^ mice were created and used to induce the diabetes mellitus type 2 (T2DM) in order to explore its potential protective effect on DCM. The knock-in efficiency and specificity of DICAR were verified by qRT-PCR. As shown in Supplementary Fig. S3a, DICAR was overexpressed, but its parent *Tulp4* mRNA was not affected (Supplementary Fig. S3b) in the *DICAR*^*Tg*^ mice. Echocardiographic assessment of cardiac function was performed on *DICAR*^*Tg*^ mice. When compared with that from the non-diabetic wild type (WT) group, the cardiac function was impaired in wild-type diabetic (WT-DM) group as shown by parameters including the left ventricular internal diastolic diameter (LVIDd), left ventricular internal diameter in systole (LVIDs), FS, SV, LVESV, LVEDV, and CO. Clearly, the impaired cardiac function was significantly improved in diabetic *DICAR*^*Tg*^ mice (Fig. 3a). These data suggest DICAR might be a key circRNA in cardiac protection in DCM. In addition, when compared with WT mice, WGA staining revealed that the cardiomyocyte size was increased in WT-DM mice, but the increased cell size was markedly inhibited in diabetic *DICAR*^*Tg*^ mice (Fig. 3b). A similar effect of DICAR overexpression was noted in terms of the collagen deposition pattern in myocardial interstitium by Masson staining (Fig. 3c) and of the expression pattern of collagen III by immunohistochemistry (Fig. 3d). These data suggest DICAR might be a key circRNA in cardiac protection during the process of DCM.

We then determined the pyroptosis of heart tissues by the activation of GSDMD, NLRP3, caspase-1, and ASC. The results showed that these pyroptosis-related proteins were significantly inhibited in heart tissues from *DICAR*^*Tg*^ mice with T2DM compared with these from the wild-type control mice with T2DM (Fig. 3e). We also overexpressed DICAR in cultured mouse cardiomyocytes through the transfection with AD-DICAR (adenovirus-expressing DICAR expression) for 48 h followed by treatment with AGEs (200 µg/mL) for 48 h in vitro. Western blotting displayed that the effects of AGEs on the activation of GSDMD, NLRP3, caspase-1, and ASC were inhibited by DICAR overexpression in these mouse cardiomyocytes (Fig. 3f).

### Interaction between VCP and DICAR regulates pyroptosis of cardiomyocytes induced by diabetes

**Fig. 4:**
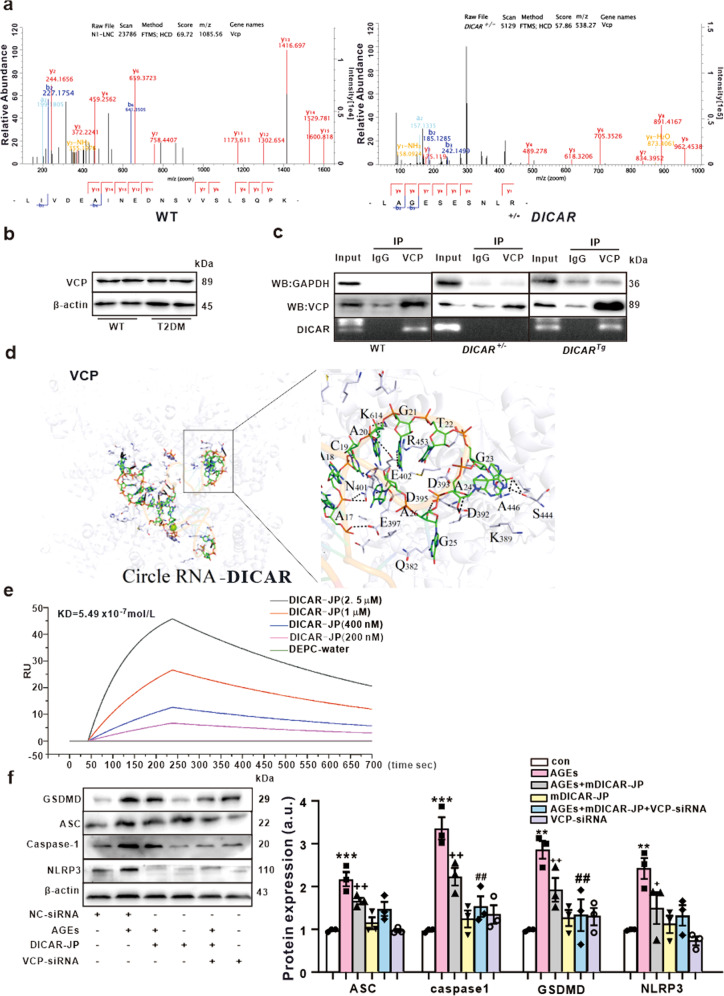
Interaction between VCP and DICAR in the pyroptosis of cardiomyocytes induced by AGEs. **a** Representative images of VCP pulled down by DICAR probe and detected by CHIRP-MS. **b** Total VCP protein expression between WT and db/db mice. **c** RIP used to detect the combination between VCP and DICAR. **d** Model prediction of VCP combined with DICAR. **e** the KD of DICAR combined with VCP detected by SPR. **f** the effect of VCP-siRNA on anti-pyroptosis induced by AGEs. Data are represented as mean ± SEM. ^**^P < 0.01, ^***^P < 0.001 vs. con; ^++^*P* < 0.01 vs. AGEs (200 µg/ml, 48 h); ^##^*P* < 0.01 vs. AGEs +mDICAR-JP, *n* = 4

Bioinformatics’ analysis of the second structure of DICAR revealed a specific stem loop structure in the junction domain (Fig. 1I). As we found that DICAR located in the cytoplasm of cardiac cells (Fig. 1g), we determined whether DICAR could interact and regulate proteins. Chromatin isolation by RNA purification-mass spectrometry (ChIRP-MS), a method used to detect the binding proteins to ncRNA, was used to identify the binding proteins of DICAR.The biotin-labeled probe of the DICAR junction site was purified and subjected to the circRNA pull-down assays by incubation with mouse heart tissue lysates, followed by MS detection. A total of 50 proteins were detected (Supplementary Table S2), among them the valosin-containing protein (VCP) had the highest score based on unique peptides and sequence coverage (Supplementary Table S2). VCP, also known as transitional endoplasmic reticulum (tER) ATPase or p97, is an abundant and highly conserved member of the ATPases family associated with a variety of cellular activities (AAA), which could control the critical steps in the ubiquitin-proteasome (Ub-Pr) degradation pathway.^23^ DICAR-binding VCP protein was further verified by parallel reaction monitoring (PRM), and the results revealed the inhibition of its expression was up to 31% in db/db mouse hearts when compared with that in WT control hearts (Fig. 4a). However, the expression of total VCP protein was not different between the two groups (Fig. 4b). In addition, to measure the binding ability of VCP and DICAR, the RIP assay was performed with a VCP antibody, followed by qPCR for DICAR. As shown in Fig. 4c, the specificity of the IP was assessed by Western blotting, which revealed a unique VCP band detected in the VCP IP, but not in IgG IP. The amount of DICAR-binding with VCP was the highest in the *DICAR*^*Tg*^ mouse hearts and moderate in the WT mouse hearts, and undetectable in *DICAR*^*+/−*^ mouse hearts.

To further explore the DICAR-VCP binding model, we employed a complete structure-based computational framework, Rosetta–Vienna RNP-_ΔΔ_G, for predicting the DICAR-VCP relative binding affinities, so as to bring together secondary structure-based energetic calculations of unbound RNA-free energies and a unified energy function for bound DICAR–VCP complexes. DICAR mainly bound three parts of the VCP protein (Supplementary Table S3). As shown in Fig. 4d, in the docking region, the secondary RNA structure of DICAR (19 bp) is bound with the amino acid residues of the VCP protein through a hydrogen bond, salt bridge, amino acid residue side chain network, and static electricity models (Supplementary Table S3). Moreover, to validate the binding affinities of the DICAR and VCP, we synthesized a 19-bp fragment of the DICAR junction part (DICAR-JP). We measured the kinetic rate constants between DICAR and VCP by the SPR assay. The results revealed that the binding constant (Kd) of DICAR-JP was 5.4 × 10^−9^ (Fig. 4e). Thus, we demonstrated that DICAR could bind to VCP via the junction fragment and that DICAR-JP was the key fragment of DICAR for VCP binding.

To explore the role of the DICAR-JP/VCP complex in the pyroptosis of cardiomyocytes induced by diabetes, synthetic mouse DICAR-JP (mDICAR-JP), human DICAR-JP (hDICAR-JP), and VCP-siRNA were studied in vitro. As illustrated in Supplementary Fig. S4a, AGEs induced the pyroptosis of HL-1 cardiomyocytes in a dose-dependent manner. Both mouse and human DICAR-JP exhibited the best anti-pyroptosis effect at the dose of 20 nM (Supplementary Fig. S4b, c). In addition, VCP siRNA inhibited the effect of AGEs on the pyroptosis of MCM in a dose-dependent manner (Supplementary Fig. S5). To explore the combined effect of DICAR-JP and VCP, we transfected DICAR-JP and VCP-siRNA together into MCMs. As shown in Fig. 4f, DICAR-JP inhibited pyroptosis induced by AGEs and VCP-siRNA enhanced the effect of DICAR-JP on pyroptosis. Thus, the DICAR-JP/VCP complex located in cytoplasm may play a key role in the pyroptosis in DCM.

### DICAR regulates VCP-mediated Med12 protein degradation via the Ub-protein system

**Fig. 5:**
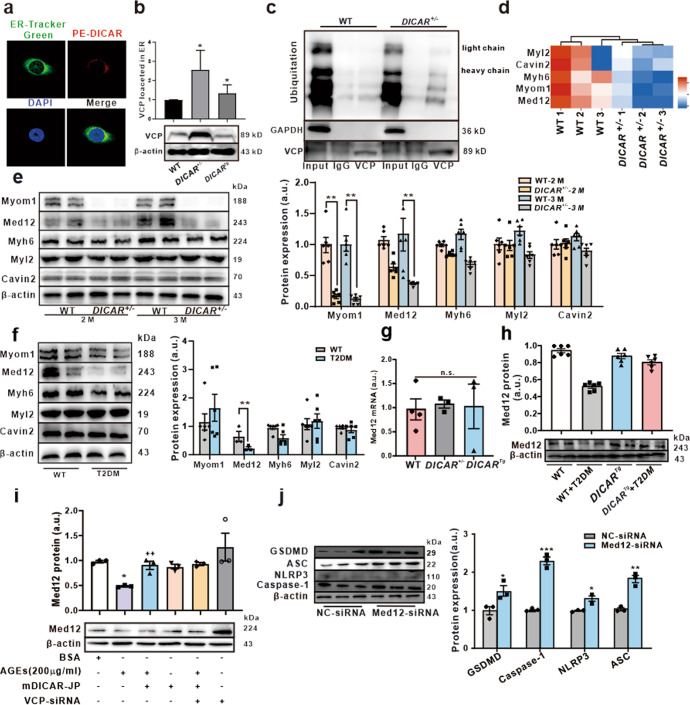
VCP mediated Med12 protein degradation via the Ub-protein system. **a** The FISH probe detected DICAR located in the endoplasmic reticulum. **b** VCP expression in ER, ^*^P < 0.05 *vs*. WT, *n* = 3. **c** Co-IP was used to detect the ubiquitination level of VCP in the heart tissues of *DICAR*^*+/−*^ mice. **d** LC-MS + PRM was used to detect the protein expression in the heart tissues of *DICAR*^*+/*−^ mice. **e** Myom1, Med12, Myh6, Myl2, and Cavin2 protein expression in the heart tissues of *DICAR*^*+/−*^ mice detected by WB. **f** Myom1, Med12, Myh6, Myl2 and Cavin2 protein expression in the heart tissues of db/db mice detected by WB. Data are represented as mean ± SEM. ^**^P < 0.01 *vs*. WT, *n* = 8. **g** Med12 mRNA in the heart tissues of DICAR + /− and DICARTg detected by qPCR. **h** Representative images of WB detected pyroptosis of the heart in the *DICAR*^*Tg*^ mouse treated by T2DM. Data are represented as mean ± SEM. ^***^P < 0.001 *vs*. WT, *n* = 8. **i** Representative images of DICAR-JP and VCP siRNA effect on the Med12 protein expression. Data are represented as mean ± SEM. ^**^P < 0.01 *vs*. BSA, ^++^*P* < 0.01 vs^.^ AGEs (200 µg/mL, 48 h), *n* = 3^.^ **j** Representative images of Med12 siRNA-induced pyroptosis of HL-1. Data are represented as mean ± SEM. ^*^P < 0.05, ^***^P < 0.01, ^***^P < 0.001, vs. NC-siRNA, *n* = 3

VCP is involved in the formation of the tER and acts as a chaperon to export misfolded proteins from the ER to the cytoplasm, where the ubiquitinated proteins are degraded via the proteasome.^24–26^ We thus hypothesize that DICAR-JP may target VCP located in the ER to exert its function. To test it, the FISH assay was performed to detect whether DICAR was marked by the PE-DICAR probe located in the ER, which was shown with the ER-tracker Green (Fig. 5a). We also tested the DICAR regulation of VCP located in the ER. When compared with that in the WT group, *DICAR*^*+/−*^ promoted the VCP expression in the ER and *DICAR*^*Tg*^ inhibited VCP located in the ER (Fig. 5b). However, DICAR-overexpression could not reduce the VCP level in the ER to the level lower than that under normal physiological condition. We then tested whether DICAR could mediate the ubiquitination of protein by interacting with VCP during ubiquitinated protein degradation. The ubiquitination levels of proteins combined with VCP were measured in hearts. Interestingly, we found that the protein ubiquitination level was downregulated in *DICAR*^*+/−*^ mice (Fig. 5c). We further confirmed that the special sequence of the DICAR junction site is a molecular chaperone of ubiquitinated protein, which could assist in the ubiquitination degradation of the proteins. AGEs could downregulate DICAR, which in turn promoted the VCP function in the development of cardiac pyroptosis in diabetes. The junction site of DICAR may act as the key regulator of cardiomyocyte pyroptosis.

Based on the functions of VCP in protein degradation and its interaction with DICAR, the LC-MS was performed to detect the protein level of the heart tissues between WT and *DICAR*^*+/*−^ mice (Fig. 5d). As shown in Fig. 5d, five proteins were downregulated in the ubiquitination process, including Myom1, Myh6, Med12, Myl2, and Cavin2. We then detected the expressions of these proteins in the heart tissues from *DICAR*^*+/−*^ mice. As shown in Fig. 5e, among the 5 proteins, Med12 was the most downregulated proteins in mouse hearts with *DICAR*^*+/−*^. In order to find whether the level of Med12 mRNA was also regulated, we detected the mRNA of Med12 in *DICAR*^*+/−*^ and *DICAR*^*Tg*^ mouse model. As shown in Fig. 5f, Med12 mRNA was not changed. These five proteins were detected in the heart of T2DM mouse, in which Med12 was also the most downregulated proteins (Fig. 5g). The results suggested that downregulation of DICAR might relieve VCP to promote Med12 protein deregulation via the Ub-protein degradation system.

It has been reported that Med12 is involved in the differentiation of endothelial cells.^27,28^ We found that the Med12 expression was decreased in T2DM hearts, which was upregulated in the hearts from diabetic *DICAR*^*Tg*^ mice (Fig. 5h). As the results shown in Fig. 5i, mDICAR reversed the dowregulated effect of AGEs (200 µg/mL, 24 h) on Med12 protein. However, co-transfected with DICAR-JP and VCP-siRNA did not play better effect on reversed Med12 expression compared with DICAR-JP (Fig. 5i). This was the limitation that VCP-overexpression plasmid be transfected into the cell to explore the block function on DICAR-JP. In addition, we had successfully founded Med12 expression interrupted HL-1 cell model via Med12 siRNA (Fig. S6). Furthermore, Med12 siRNA alone could also induce pyroptosis in the HL-1 cardiomyocytes (Fig. 5j).

### The levels of DICAR in PBMCs and in plasma are decreased in diabetic patients

To provide a clinical link between DICAR expression and diabetes in patients, we determined the levels of DICAR in peripheral blood mononuclear cells (PBMCs) and in circulating plasma from three age-matched human groups: normal heath control subjects, the patients with diabetes (T2DM) with and without cardiac dysfunction. The gender, age, HR, BMI, SBP, DBP, Cr, and BUN were not different among the three groups. TG, TC, and HG were higher in patients with diabetes (Table 1). DICAR level in healthy individuals was 1.90 ± 0.34 in PBMCs and 1.22 ± 0.32 in plasma, respectively, while it was decreased in diabetic patients with cardiac dysfunction (PBMCs: 0.84 ± 0.12; plasma: 0.88 ± 0.17) and in diabetic patients without cardiac dysfunction (PBMCs: 0.39 ± 0.06; plasma: 0.57 ± 0.10) (Table 1).

**Table 1:** Clinical information and DICAR expression in diabetic patients

Groups	Healthy (*n* = 12)	T2DM (*n* = 21)	T2DM with HDF (*n* = 9)
Age	68 ± 10	65 ± 9	68 ± 12
HR (/min)	81 ± 12	81 ± 8	82 ± 12
BMI (kg/m^2^)	23.1 ± 1.2	23.8 ± 1.3	23.9 ± 1.5
SBP (mmHg)	123.0 ± 11.2	124.0 ± 10.9	125.0 ± 10.3
DBP (mmHg)	70.0 ± 10.9	72.0 ± 10.7	71.0 ± 11.2
Cr (μmol/L)	72.83 ± 15.78	66.14 ± 20.43	76.32 ± 25.91
BUN (mmol/L)	4.75 ± 0.92	4.94 ± 1.51	5.38 ± 1.63
TC (mmol/L)	2.94 ± 2.26	4.79 ± 0.82^**^	3.96 ± 1.17^*^
TG (mmol/L)	0.85 ± 0.77	1.46 ± 0.60^**^	1.52 ± 1.14^**^
HDL-C (mmol/L)	1.6 ± 0.1	1.17 ± 0.33^*^	1.1 ± 0.2^*^
FBG (mmol/L)	5.4 ± 0.6	8.8 ± 1.0^**^	8.5 ± 1.5^**^
EF (%)	54.64 ± 0.92	55.33 ± 0.63	36.08 ± 3.22^***^
DICAR in PBMC (a.u.)	1.90 ± 0.34	0.84 ± 0.12^***^	0.88 ± 0.17^***^
DICAR in plasma (a.u.)	1.22 ± 0.32	0.39 ± 0.06^**^	0.57 ± 0.10^**^

*HDF* heart dysfunction, *HR* heart rate, *BMI* body mass index, *SBP* systolic blood pressure, *DBP* dilated blood pressure, BUN, *TC* total cholesterol, *TG* triglyceride, *HDL* high density lipoprotein-C, *FBG* fasting blood glucose, *EF* ejection fraction. *P *<* *0.05*, **P *<* *0.01*, ***P *<* *0.001* vs. Healthy individials, *n* = 9–21

## Discussion

Our study has identified a novel circRNA, which is decreased in diabetic hearts and is involved in the development of DCM. Based on its biological functions, we named it as DICAR. DICAR has an inhibitory effect on the development of DCM, as the spontaneous cardiac dysfunction, cardiac cell hypertrophy, and cardiac fibrosis occur in heterozygous DICAR deficiency (*DICAR*^*+/−*^) mice. In contrast, the DCM is alleviated in DICAR-overexpressed *DICAR*^*Tg*^ mice. In addition, the homozygous DICAR deficiency (*DICAR*^*−/−*^) is lethal to mice, which suggests that DICAR is indeed a critical circRNA at least in mice.

The pyroptosis of cardiomyocytes is a recently identified key cellular event in the development of DCM.^29^ To uncover the potential cellular mechanisms involved in DICAR-mediated effect on DCM, the pyroptosis of cardiomyocytes has been determined via both gain-of-function and loss-of-functional approaches in DICAR gene-modulated mice in vivo and in cultured cardiomyocytes in vitro. The results have demonstrated that DICAR overexpression inhibits pyroptosis of cardiomyocytes in T2DM in vivo and in AGEs-treated cardiomyocytes in vitro, whereas DICAR knockdown has an opposite effect on cardiomyocyte pyroptosis. The effect of DICAR on cell apoptosis, another cell damage in DCM should also be determined in future studies. At the molecular level, we have found that DICAR downregulation under diabetic condition is able to release VCP and then mediates the Med12 protein degradation via the Ub-Pr system, which in turn could induce pyroptosis of cardiomyocytes during the development of DCM.

In this study, we determined the effects of DICAR deficiency (*DICAR*^*+/−*^) on cardiac dysfunction, cardiac cell hypertrophy and cardiac fibrosis, as well as on cardiac cell pyroptosis every month up to 5 months after birth. We found that the damages of cardiac function, cardiac cell hypertrophy and cardiac fibrosis in *DICAR*^*+/−*^ mice reached the peak at 3 months old. No additional deterioration was found at 4 and 5 months old. For the cardiac cell pyroptosis, we found that it reached the peak at 2 months old. We thought that in the early stage when the *DICAR*^*+/−*^ mouse was born, DICAR deficiency could induce cardiac cell pyroptosis and secrete inflammatory factors. Those cellular responses would gradually cause the damages of cardiac function and cardiac structures. However, in response to DICAR deficiency-induced cardiac cell pyroptosis, the cardiac cells may evoke some compensatory responses to against pyroptosis which made the cell pyroptosis to reach a peak at 2 months old. However, the damages of cardiac function and cardiac structures induced by cellular injury responses might reflect the delayed results, which reached a peak at 3 months old.

CircRNAs have more stable structure with less immunogenicity compared with the linear RNAs, which makes them have great potential for the new drug candidates.^30^ Based on the closed structure of DICAR, we have identified that its junction site forms a specific loop RNA second structure which makes its structure more stable even though DICAR is degraded. Also, the junction site sequence of DICAR is different part between DICAR and its parent line mRNA. We further identified that the 19-bp fragment of the DICAR-JP is a critical functional part of DICAR. Interestingly, we do find the synthesized DICAR-JP has a therapeutic effect on diabetes-related pyroptosis of cardiomyocytes. Therefore, DICAR-JP could be a novel small RNA which may be a good candidate for nucleic acid drugs in diabetes.

Recent studies have reported that circRNAs could regulate diseases and that circRNAs can be considered as potential markers for the diagnosis of several diseases as well as therapeutic target genes.^31^ For example, circRNA HRCR is proposed to possess the ability to sponge miR-223 and is beneficial for cardiac hypertrophy and heart failure.^32^ In addition, circ-Foxo3 is found to be able to promote cardiac senescence.^33^ To date, few studies have been performed to test the role of circRNAs in DCM. This study provides new information in this research field by identifying the role of the circRNA DICAR in the development of DCM. It is clear that DICAR-mediated biological functions are by DICAR itself, which is not related to its parent gene, as in both *DICAR*^*+/*−^ and *DICAR*^*Tg*^ mice, the parent gene *Tulp4* expression remains unchanged. DICAR itself may be an important target for the treatment of DCM.

Pyroptosis, a new type of programmed cell death, was proven to be activated in DCM. Alleviating pyroptosis exerts a beneficial effect on DCM by interrupting the long ncRNA Kcnq1ot1 expression. NLRP3 is a key protein that mediates pyroptosis and *NLRP3* knockdown could ameliorate DCM in type 2 diabetes.^12,34^ In the current study, we found that DICAR knockdown could induce pyroptosis as well as activate NLRP3 in mouse hearts. In *DICAR*^*Tg*^ T2DM mice, pyroptosis is inhibited compared with that in WT mice with T2DM. In cultured cardiomyocytes, pyroptosis is activated by AGEs and that the AGEs-induced pyroptosis is reversed via DICAR overexpression. The DICAR can be considered as a key regulator of pyroptosis in diabetes. One weakness of our pyroptosis study was that the determination was mainly by western blot analysis of the pyroptosis-related proteins. Other methods such as live-cell imaging should be applied to further verify the pyroptosis results.

One of the mechanisms of circRNA’s function is sponging miRNAs. For example, caspase-1 is regulated by hsa_circ_0076631 by targeting miR-214-3p in DCM. More recently, circRNAs may act through other diverse mechanisms in addition to miRNA sponging, which include sequestering proteins, regulating gene transcription, and even providing a protein synthesis template.^15^ Our current research reveals that the DICAR could bind with VCP via the DICAR-JP at the junction site and modulates the action of VCP. DICAR expression is decreased and the effect of VCP on the degradation of the ubiquitinated protein is thus enhanced in diabetes.

VCP disruption of valosin-containing protein activity causes cardiomyopathy and reveals pleiotropic functions in cardiac homeostasis.^35^ In patients with acute coronary syndrome (ACS) group, the serum VCP levels were significantly higher than the normal groups, which could be used as a stable biomarker in predicting the development of ACS and its ventricular dysfunction (VD).^36^ Many cofactors can interact with VCP that regulate its function by recruiting VCP to different cellular pathways.^37^ Most of the cofactors bind the N-terminal of VCP, while several others bind to the C-terminal. All the previous research reports regarding the VCP binding are based on protein interaction.^38^ In the current study, we have identified, for the first time, that the secondary structure of a circRNA (DICAR) junction site acts as a functional domain motif to bind to proteins such as VCP.

Med12, a subunit of the RNA polymerase II transcription, acts as a key mediator in many biomedical processes.^34^ Previous studies have reported that Med12 is able to regulate several key signaling pathways involved in cell growth, development, and differentiation.^39^ Recently, one study has found that in mice with conditional cardiac-specific knockout of Med12, the mouse hearts display progressively dilated dysfunction, which suggests that Med12 is also a key molecule in maintaining normal cardiac functions.^40^ In this study, we have found that Med12 is downregulated in hearts from T2DM mice and from *DICAR*^*+/*−^ mouse. In addition, Med12 is involved in the development of pyroptosis of MCMs. The results suggest that DICAR/VCP is able to regulate the Med12 degradation process and may mediate the pyroptosis of cardiomyocytes in DCM. It will be better to perform a rescue experiment to check whether Med12 overexpression could rescue the phenotype of pyroptosis and cardiac dysfunction of *DICAR*^*+/−*^ mice to further confirm the role of Med12.

Heart tissue sample of DCM is very difficult to be obtained, especially from diabetic patients without serious heart failure and other serious complicated diseases. To provide an alternative clinical link of DICAR with diabetes, we have measured the levels of DICAR in circulating blood cells (PBMCs) and in plasma from diabetic patients and their health controls. In PBMCs and in plasma, the levels of DICAR are significantly lower in diabetic patients than those in health controls, which were consistent with the DICAR downregulation in diabetic mouse hearts. However, we have not found difference of DICAR expression in PBMCs and in plasma between patients with (*n* = 9) and without (*n* = 21) cardiac dysfunction. The results suggested that downregulation of DICAR might not be limited to cardiac cells and hearts under diabetic conditions. In addition, the circulating plasma has low level of DICAR, which indicates that the cells may release some of their DICAR into the extracellular space and circulating blood. DICAR in PBMCs and in plasma might be a novel biomarker for diabetes. We did not find difference between diabetic patients with and without cardiac dysfunction. We thought there were two possibilities. First, the case number of diabetic patients with cardiac dysfunction was small. The increasing of case number in each group will be required to confirm the discovery in diabetic patients. The second possibility was that, although the decrease in DICAR expression may be a general phenomenon in cells and plasma from diabetic patients, the organ tissue expression levels might be a better parameter to reflect the specific organ damages induced by diabetes. Thus, the expression levels of DICAR in heart tissues from diabetic patients should be measured in future studies.

In summary, our study has identified that the novel circRNA DICAR, which is down-regulated in diabetes, could protect against diabetes-induced pyroptosis of cardiomyocytes and the development of diabetic cardiomyopathy. The underlying molecular mechanisms are the binding of the DICAR-junction domain with VCP and regulate the pyroptosis by VCP-mediating Med12 degradation through the ubiquitin-proteasome (Ub-Pr) pathway . DICAR and the synthesized DICAR-JP may be good candidates for novel nucleic acid drugs in the prevention and treatment of diabetic cardiomyopathy.

## Materials and methods

### Patient samples

To explore the relationship with the DICAR expression and the cardiac dysfunction induced by diabetes, we collected the healthy individuals (*n* = 12), the age was between 50–70; T2DM patients without cardiac dysfunction or any other complication (*n* = 21) and T2DM patients with cardiac dysfunction (*n* = 9). All the patients enrolled in this study had no extracardiac complications and were from China Resource & WISCO General Hospital. The study was conducted according to the standards of the declaration of Helsinki. The study was approved by the Ethics Committee of China Resource & WISCO General Hospital. Written informed consent was obtained from all subjects.

### Reagents and antibodies

All the reagents and antibodies were listed in the supplementary key resources table.

### Animals

C57BL/KsJ WT (age: 12 weeks) and C57BL/KsJ db/db mice of both genders and with 23–28 (mM) blood glucose levels were purchased from the breeding colonies of GenePharmatech Company. *DICAR*^*+/−*^ *and DICAR*^*Tg*^ mice were all established at the Model Animal Research Center of Nanjing University, China (see the Results section). All animal studies were conducted with age- and gender-matched controls and they were maintained in a temperature-controlled (22–25 °C) environment under a 12-h light/dark cycle and free access to food and water at the Animal Center Wuhan University of Science and Technology.

### Adenoviral constructions and infection

To construct the adenoviruses, circRNA mm9-circ-008009 (renamed as DICAR) is a vector synthesized by Chenechem Company (Shanghai, China). DICAR was first inserted into pcDNA3.1 with the endogenous flanking sequence (1-kb upstream). The upstream flanking sequence was then partially copied and then inserted in the inverted orientation downstream. Adenovirus-DICAR-shRNA without the downstream reverse sequence served as the negative control. All these vectors were finally cloned into the Adeno-X TM Expression System (Clontech) in accordance with the manufacturer’s instructions.^41^

### Microarray analysis

CircRNA microarray analysis was performed using the Arraystar mouse V.2 (Kangcheng Biotechnology Company, Shanghai). Total RNA from each heart tissue sample was quantified using the NanoDrop ND-1000 system. The sample preparation and microarray hybridization were performed based on Arraystar’s standard protocols. Total RNAs were digested with RNAse R (Epicentre, Inc.) to remove linear RNAs and enrich circRNAs. Then, the enriched circRNAs were amplified and transcribed into fluorescent cRNA by using a random priming method (Arraystar Super RNA Labeling Kit; Arraystar). The labeled cRNAs were hybridized onto the Arraystar Mouse circRNA Array (8 x 15 K, Arraystar). After washing the slides, the arrays were scanned by the Agilent Scanner G2505C. Agilent Feature Extraction software (version 11.0.1.1) was used to analyze the acquired array images. Quantile normalization and subsequent data processing were performed using the R software package. Finally, the differentially expressed circRNAs with statistical significance between the two groups were identified through Volcano Plot filtering. The differentially expressed circRNAs between the two samples were identified through fold-change filtering. Hierarchical clustering was performed to demonstrate distinguishable circRNAs expression patterns among the samples.

### RNase R treatment

Total RNA was extracted from mouse heart tissues and classified into two groups. One group of RNA was pretreated with RNase R (Epicentre, USA), 3 U/µg RNA at 37 °C for 15 min, according to the manufacturer’s instructions. The other group served as control. Then, qRT-PCR was performed to detect the expression of mm9-circ-008009, hsa_circ_0131202 and GAPDH with/without RNase R treatment.

### Immunofluorescence assays and FISH

Immunofluorescence assays were performed as described in the previous study.^42^

Cy3-labeled DICAR, FITC-labeled TnnT were used as described by the manufacturer’s instructions.^42^ FL glibenclamide was used to mark the ER. The live cells were stained by FL Glibenclamide and fixed by the DICAR FISH probe A. The FV10i Confocal Microscope (Olympus, Japan) was used to capture the images.

### The peripheral monocyte isolation from diabetes patients with or without cardiac dysfunction

The blood samples were processed within 1 h of blood sampling. Every patient signed a specific informed consent form, and clinical data were collected from the medical reports and transferred to an anonymous database for statistical processing. Peripheral blood samples of patients and healthy donors were collected using ethylenediaminetetraacetic acid (EDTA) anticoagulated vacutainers (BD Biosciences). Before separation, blood was mixed with the same volume of PBS and then overlaid on a FicollHypaque gradient (1077 g/mL; Cedarlane, Cat CL5020). Density centrifugation was performed at room temperature at 1800 rpm for 30 min with no brake. Peripheral blood mononuclear cells (PBMCs) were collected and stored at −196 °C [(1 × 10^7^ cells in 10% DMSO + 90% fetal calf serum (FCS)]. The study was conducted according to the standards of the Declaration of Helsinki. The study was approved by the Ethics Committee of China Resource & WISCO General Hospital. Written informed consent was obtained from all subjects.

### DICAR-VCP testing for binding affinity prediction

Rosetta-Vienna RNP-^ΔΔ^ was employed in this study by combining the 3D structure modeling with RNA secondary structure-based energetic calculations in order to predict the RNA-protein relative binding affinities. Briefly, a complete calculation framework for RNA–protein binding affinities was referred to using bioinformatics that included a unified free-energy function for bound complexes and automated Rosetta modeling of mutations. The secondary structure-based energetic calculations were performed to model the unbound RNA states. Calculating the energy relationship between a series of VCP proteins and DICAR revealed that the mode of action between DICAR and VCP proteins was interpreted and ranked by energy. Finally, the DICAR with a better binding ability to VCP was selected in accordance with the energy score.

### Statistical analyses

All data are presented as means ± SEM. Statistical analysis for the comparison of two groups was performed using a two-tailed unpaired Student’s *t*-test. To compare more than two groups, a one-way analysis of variance (ANOVA), followed by Tukey’s post hoc test was performed. Adjusted two-sided *P*-values were calculated, and *P* < 0.05 was considered to indicate statistical significance. All statistical analyses were performed with the GraphPad Prism Version 6 (GraphPad Software Inc., SanDiego, CA, USA) and SPSS package (SPSS Inc., Chicago, IL, USA).

## Supplementary information

Supplementary materials

orignial wb picutre

## Data Availability

The datasets generated and/or analyzed during the current study are available from the corresponding author upon reasonable request. All data needed to evaluate the conclusions in the paper are present in the paper and/or the Supplementary Materials. The high throughput raw and processed data have been deposited in GEO (http://www.ncbi.nlm.nih.gov/geo/query/acc.cgi?acc=GSE199133). The ubiquitin remnant motif mass spectrometry proteomics data have been deposited to the ProteomeXchange Consortium via the PRIDE partner repository with the dataset identifier PXD037732. The CHIRP-MS proteomics data have been deposited to the iProxX intergrated proteome resources with the dataset identifier PXD037745.
