# Supplementary material for: CircRNA DICAR as a novel endogenous regulator for diabetic cardiomyopathy and diabetic pyroptosis of cardiomyocytes
Source: Signal Transduct Target Ther. 2023 Mar 8;8:99. doi: 10.1038/s41392-022-01306-2 (PMC9992392; doi:10.1038/s41392-022-01306-2)
Supplement: Supplementary file 1 — Supplementary materials [file 41392_2022_1306_MOESM1_ESM.docx]

Supplementary Materials for

CircRNA DICAR as a novel endogenous inhibitor for diabetic cardiomyopathy and diabetic pyroptosis of cardiomyocytes

Qiong Yuan^1^^,2+^, Yunwei Sun^2+^, Fan Yang^3+^, Dan Yan^2^, Meihua Shen^2^, Zhigang Jin^4^, Lin Zhan^2^, Guangqi Liu^2^, Ling Yang^2^, Qianyi Zhou^2^, Zhijun Yu^2^, Xiangyu Zhou^1^, Yang Yu^1^, Yong Xu^1^, Qingming Wu^2^, Jianfang Luo^6^, Xiamin Hu^5^*, Chunxiang Zhang^1^*

^1^Department of Cardiology, the Affiliated Hospital of Southwest Medical University and Key Laboratory of Medical Electrophysiology, Ministry of Education, Institute of Cardiovascular Research and Institute of Metabolic Diseases, Southwest Medical University, Luzhou 646000, China

^2^College of Medicine, Wuhan University of Science and Technology, Wuhan, 430065, China.

^3^Department of Emergency and Critical Care Medicine, Guangdong Provincial People’s Hospital, Guangdong Academy of Medical Sciences, Guangzhou, 510080, China

^4^China Resource & WISCO General Hospital, Wuhan University of Science and Technology, Wuhan, 430065, China

^5^College of Pharmacy, Shanghai University of Medicine and Health Sciences, Shanghai, 210000, China.

^6^Department of Cardiology, Guangdong Cardiovascular Institute, Guangdong Provincial Key Laboratory of Coronary Heart Disease Prevention, Guangdong General Hospital, Guangdong Academy of Medical Sciences, Guangzhou, 510100, China.

*Correspondence: Chunxiang Zhang. zhangchx999@163.com, Key Laboratory of Medical Electrophysiology, Ministry of Education, Institute of Cardiovascular Research, Southwest Medical University, Luzhou 646000, China; Xiamin Hu, Email: [huxiaming@163.com](mailto:huxiaming@163.com), College of Pharmacy, Shanghai University of Medicine and Health Sciences, Shanghai, 201318, China;.

^+^Contributed equally to this work and should be considered joint first authors.

**This PDF file includes:**

Supplementary Methods

Key Resources Table

Figure S1 to S6

Table S1 to S4

**Supplementary Methods:**

**Cardiomyocyte culture**

First, C57BL/6 mouse cardiomyocytes were isolated from 1–2-day-old mice. The mouse heart was removed and excised into pieces of size approximately 1 mm. The tissues were digested with trypsin and type-II collagenase. The cells were plated in 10-μg/mL laminin-coated different culture dishes, and the cardiomyocytes were collected after differential adherence. The cardiomyocytes were incubated in a 37°C incubator after seeding in culture plates, followed by 24 h of serum starvation. At the end of incubation, the cells were either infected with adenoviruses at a specific multiplicity of infection (MOI) or with other treatments. The mouse cardiomyocyte cell line HL-1 was cultured in the DMEM medium. The HCM cell line was sourced from Abm (Richmond, Canada) and cultured in PriGrow I (Abm).

**qPCR reaction analysis**

The mRNA expression of circRNAs were analysed using the ABI 7300 Real-Time PCR System (Foster City, CA, USA). The specifific primer pairs are shown in supplymentary Table S5. Reverse transcription reaction was performed with 1 μg total RNA isolated from the cells of each group. For qPCR amplifification, cDNAs were amplifified using the SYBR Green Real-Time PCR Master Mix (Takara) and 0.4 μmol/L of each primer pair. All the primers were shown in Table S5. Amplifification was carried out starting with an initial step for 30 s at 94^o^C, followed by 40 cycles of the amplifification step (94^o^C 30 s, 60^o^C 60 s and 72 ^o^C 1 min). All amplifification reaction for each sample was carried out in triplicate and the averages of the threshold cycles were used to interpolate curves using 7300 System SDS Software (ABI, CA, USA). Results were expressed as the ratio of circRNAs/GAPDH expression level in the group of the control was regarded as 100%.

**Echocardiography of cardiac dimensions and functions**

The cardiac function was detected by echocardiography. The use of inhaled isoflurane helps lesser trained operators to achieve physiological images. The use of a uniform dosage of isoflurane throughout the experiment minimizes the measurement errors. Different mouse strains show varying susceptibility to isoflurane. In this study, the isoflurane dosage was specifically tailored to each mouse strain. Echocardiography was performed for the *in vivo* assessment of cardiac structures and functions. Relevant measurements included left ventricular wall thickness, internal dimension, mass, and EF. After isoproterenol treatment, re-evaluation was performed by conducting an echocardiogram as frequently as desired. We monitored the mice monthly and recorded the two-dimensional-guided M-mode tracings in both parasternal long-axis and short-axis views at the level of the papillary muscles. The LV wall thickness and LVIDd were accordingly measured. FS was calculated with the established standard equation. All measurements were performed from more than three beats and then averaged.

**Fluorescence *in situ* hybridization (FISH)**

The mouse heart tissues, MCM, and HCM were fixed with 4% paraformaldehyde and prehybridized and hybridized in a hybridization buffer. All mouse DICAR and human DICAR-targeted FISH probes were designed according to the junction region of DICAR and synthesized by the Ruibo Biotechnology Co., Ltd. (Guangzhou, China). The signals of the probe were detected by the FISH Kit (Ruibo Biotechnology) according to the manufacturer’s instructions. Nuclei were stained with DAPI and the images were captured on a confocal microscope.

**Type 2 diabetes mellitus mouse model**

The WT and *DICAR^Tg^* mouse were fed with west diet for three months and tail vein injection with alloxan monohydrate (50 mg/kg body weight; Sigma-Aldrich, USA). The animals with blood sugar level of ≥200 mg/dl were considered diabetic and were used in the current investigation. Blood glucose levels were measured by the On Call EZ II blood glucose monitoring system (ACON Biotech, USA). The biochemical analyzer (ERBA Chem., India) and commercial ERBA diagnostic kits were used for serum analysis of total cholesterol (TC), triglyceride (TG), high-density lipoprotein-cholesterol (HDL-C), and low-density lipoprotein-cholesterol (LDL-C).

**Western blotting**

The expression levels of several proteins were detected by Western blotting. Briefly, the heart tissues and cells were homogenized in the RIPA lysis buffer (Beyotime, Jiangsu, China) supplemented with 0.1 mM phenylmethylsulfonylfluoride (PMSF) (Sigma, Missouri, USA) for immunoblotting analysis. The cells were harvested and lysed in a lysis buffer. The proteins were separated using sodium dodecyl sulfate-12% polyacrylamide gel electrophoresis (SDS-PAGE) and transferred onto a polyvinylidene fluoride (PVDF) membrane at 300 mA for 1.5 h. Subsequently, the membrane was incubated in TBS/T buffer (20 mM Tris-HCl, pH 7.6, 150 mM NaCl, 0.1% Tween-20) with 5% non-fat milk at room temperature for 2 h. Specific primary antibodies included-rabbit anti-NLRP3(D4D8T) (1:1000, Cell Signaling Technology, USA), GSDMD, ASC, mouse anti-caspase-1 (1:1000, Santa Cruz Biotechnology, USA), mouse VCP (1:1000, Santa Cruz Biotechnology), and mouse anti-β-actin (1:1000, Proteintech, Wuhan, USA); GSDMD (1:1000, Santa Cruz Biotechnology); all antibodies were diluted in TBST buffer (50 mM Tris-HCl, 150 mM NaCl, 0.1% Tween-20, pH 7.4) and incubated with the PVDF membrane at 4ºC overnight. Corresponding horseradish peroxidase (HRP)-conjugated secondary antibodies (1:5000, A21010, Abbkine, CA, USA) were subsequently incubated with the PVDF membrane for 90 min at room temperature. The signal was detected with an enhanced chemiluminescent (ECL) reagent (Amersham Biosciences, Piscataway, NJ, USA). The luminescent signals were detected by the ChemiDoc MP system (Bio-Rad).

**Histological analysis**

The hearts were excised, fixed in 10% formalin, embedded in paraffin, and sectioned into 7-μm slices. To measure the cross-sectional area of the cardiomyocytes, these sections were stained with FITC-conjugated wheat germ agglutinin (Sigma) according to the method described previously . To determine cardiac fibrosis, we stained the heart sections with the standard Masson trichrome staining according to the manufacturer’s instructions (Sigma).

**Immunohistochemistry**

For immunohistochemistry, the formalin-fixed myocardial sections were deparaffinized and rehydrated. Primary polyclonal antibody against collagen III (Rabbit polyclonal; Santa Cruze, California, USA; 1:1000 dilution). The average percentages of collagen III-stained area were calculated from 6 separate fields of transverse left ventricular tissue sections by light microscopy (× 400 magnification).

**Chromatin isolation by RNA purification–MS (CHIRP-MS)**

The heart tissues were washed with pre-cooling PBS buffer and the tissues were crosslinked with 3% formaldehyde at room temperature on an end-to-end shaker for 30 min. Quench crosslinking was performed with 125-mM glycine for 5 min, spun at 1000 RCF for 3 min, and the supernatant was discarded. The tissues were then washed twice with cooled PBS and spun at 3000 rpm. The supernatant was transferred to 2 volumes of the hybridization buffer, mixed, and incubated at 37°C. The pre-bind probe (100 pmol per 2 × 10^7^ cells) was incubated with the streptavidin beads for 30 min, the unbinding probe was washed out, and the beads were mixed with the cell lysate and hybridized at 37°C overnight on an end-to-end shaker. The beads were washed for five times with 1 mL of pre-warmed wash buffer. To the sample, 100 µL of the elution buffer, 20 U benzonase, and elute protein were added at 37°C and incubated for 1 h. The supernatant was collected and the sample was reverse cross-linked at 95°C, and the protein was precipitated with 0.1% SDS and 10% TCA at 4°C for 2 h. The sample was then spun at top speed, and the pellets were washed with pre-cold 80% acetone thrice. After the protein was digested, 1/2 peptide was separated and analyzed with the nano-UPLC (EASY-nLC1200) coupled to Q-Exactive Mass Spectrometry (Thermo Finnigan). The separation was performed on a reversed-phase column using mobile phases composed of water with 0.1% FA, 2% ACN (phase A), and 80% ACN, 0.1% FA (phase B). The sample was executed with a 120-min gradient at a 300 nL/min flow rate. The data-dependent acquisition was performed in profile and a positive mode with the Orbitrap Analyzer at the resolution of 70,000 (@200 m/z) and the m/z range of 350-1600 for MS1; For MS2, the resolution was set to 17,500 with a dynamic first mass. The automatic gain control (AGC) target for MS1 was set to 1.0^6^ with max IT 100 ms and 5.0^4^ for MS2 with max IT 200ms. The top 10 most-intense ions were fragmented by HCD with normalized collision energy (NCE) of 27% and an isolation window of 2 m/z. The dynamic exclusion time window was set to 20s.

**LC-MS/MS of ubiquitin remnant motif**

The tissue was lysed in a urea-containing buffer, the cellular proteins were digested with proteases, and the resultant peptides were purified through reversed-phase solid-phase extraction. The peptides were then subjected to immunoaffinity purification using the PTMScan^®^ ubiquitin remnant motif (K-ε-GG) antibody. The antibody was incubated with protein A agarose beads. Unbound peptides were removed through washing, and the captured ubiquitin-containing peptides were eluted with dilute acid. Reversed-phase purification was performed on microtips to desalt and separate the peptides from antibodies before concentrating the enriched peptides for LC-MS/MS analyses.

**Parallel reaction monitoring (PRM)**

The protein candidates with more than 2-fold change and adjusted P-value of <0.05 were selected for further validation through targeted liquid chromatography-parallel reaction monitoring (LC-PRM) MS. The peptides were separated on the C18-column with buffer B (90% ACN, 0.1% formic acid) at the flow rate of 300 nL/min. Mobile phase B: 6–28% for 92 min, 28–40% for 20 min, 40–100% for 2 min, 100% for 2 min, 100–2% for 2 min, 2% for 2 min. PRM MS2 spectra were collected at the resolution of 17500 with an AGC target value of 5 x 10^4^. Raw data were analyzed by Skyline using a data-independent acquisition method (version 3.6.1), with q = 0.1.

**Surface Plasmon resonance (SPR) assay**

The nucleic acid of the DICAR-junction sequence was synthesis by Sangon Biotech (Shanghai, China) Company and verified with an SPR assay using the BIAcore T200 Instrument (BIAcore T200, GE Healthcare, Chicago, IL, USA) as described elsewhere.^43^ Recombinant protein VCP was immobilized on a CM5 sensor chip, and a blank channel was employed as the negative control for each assay. A concentration of 100 µM for the selected compounds was used for the primary screening. For positive hits, the compounds were serially diluted to different concentrations with the HBS-EP + buffer and flowed through the chip. The KD values were calculated by using a steady affinity state model by the BIAcore T200 Analysis Software as described elsewhere.^44^

**RNA Immunoprecipitation (RIP) Assay**

EZ-Magna RIP™ RNA-Interacting Protein Immunoprecipitation Kit (Millipore, Billerica, MA, USA) was utilized for the RIP assay. Heart tissue underwent lysis in RIP lysis buffer with RNase inhibitor (Millipore). A 100 μL cell lysate was treated with RIP with magnetic beads coated VCP antibody. The DICAR was then precipitated prior to evaluation via RT-qPCR.

**Key Resources Table**

| Reagent or Resorce | Source | Identifier |
| --- | --- | --- |
| Trizol | Thermo Scientific Company, USA | 15596018 |
| HiScript III RT SuperMix for qPCR (+gDNA wiper) | Vazyme biotech Company, China | R323-01 |
| Hieff^®^ qPCR SYBR Green Master Mix(No Rox) | Yeasen Biotechnology, Shanghai | 11201ES08 |
| BCA Protein Assay Kit | Biosharp, China | BL521A |
| Protein loading buffer (5X) | Biosharp, China | BL502B |
| Glycine | BioFroxx, China | 1269GR500 |
| Tris-HCl | BioFroxx BioFroxx | 1115GR500 |
| BSA | Gibco, China | 11021029 |
| Mouse antibody-Caspase-1 p20 | Santa Cruz Company, USA | sc-398715 |
| Rabbit antibody-ASC | Cell signaling Technology, USA | 67824 |
| Rabbit antibody-GSDMD | Cell signaling Technology, USA | 39754 |
| Rabbit antibody-NLRP3 | Cell signaling Technology, USA | 15101 |
| HRP, Goat Anti-Rabbit IgG | Bioworde Company | bs13278 |
| HRP，Goat Anti-Mouse IgG | Bioworde Company | bs 14278 |
| Rabbit antibody-Med12 (1:1000) | Proteintech Company | 20028-1-AP |
| Rabbit antibody-Cavin2 (1:1000) | Proteintech Company | 12339-1-AP |
| Rabbit antibody-Myom1 (1:1000) | Proteintech Company | 20360-1-AP |
| Rabbit antibody-Myh6 (1:1000) | Proteintech Company | 22281-1-AP |
| Rabbit antibody-Myl2 (1:1000) | Proteintech Company | 10906-1-AP |
| Rabbit antibody-VCP (1:1000) | Cell signaling Technology, USA | 2649 |
| Rabbit antibody-ACTIN (1:1000) | Proteintech Company, China | 66009-1-Ig |
| Cy3-labeled DICAR | RiboBio, Guangzhou, China | N/A |
| FITC-labeled TnnT | RiboBio, Guangzhou, China | N/A |
| High Sensitivity ECL Kit (1:1000) | Biosharp Company, China | BL520A |
| High glucose DMEM medium (1:1000) | Gibco Company, China | C11995500 |
| Fetal bovine serum (1:1000) | Gibco Company, China | 10100147 |
| Trypsin (1:1000) | Biosharp Company, China | BL512A |
| AGEs (1:1000) | Bioss Company, China | bs-1158P |
| LV-FITC-DICAR | Genechem Company, China | N/A |
| Biological samples |  |  |
| Individuals with T2DM and healthy adult donors’ peripheral blood monocyte cells | China Resource & WISCO General Hospital, Wuhan University of Science and Technology | N/A |
| Experimental models: Cell lines |  |  |
| HL-1 | CTCC | CTCC-001-0185 |
| AC16 | Sigma-Aldrich | SCC109 |
| Experimental models: Organisms/strains | | |
| *DICAR^+/-^* | Model Animal Research Center of Nanjing University | N/A |
| *DICAR^Tg^* | Model Animal Research Center of Nanjing University | N/A |

**Supplementary Figs**

**Figure. S1**


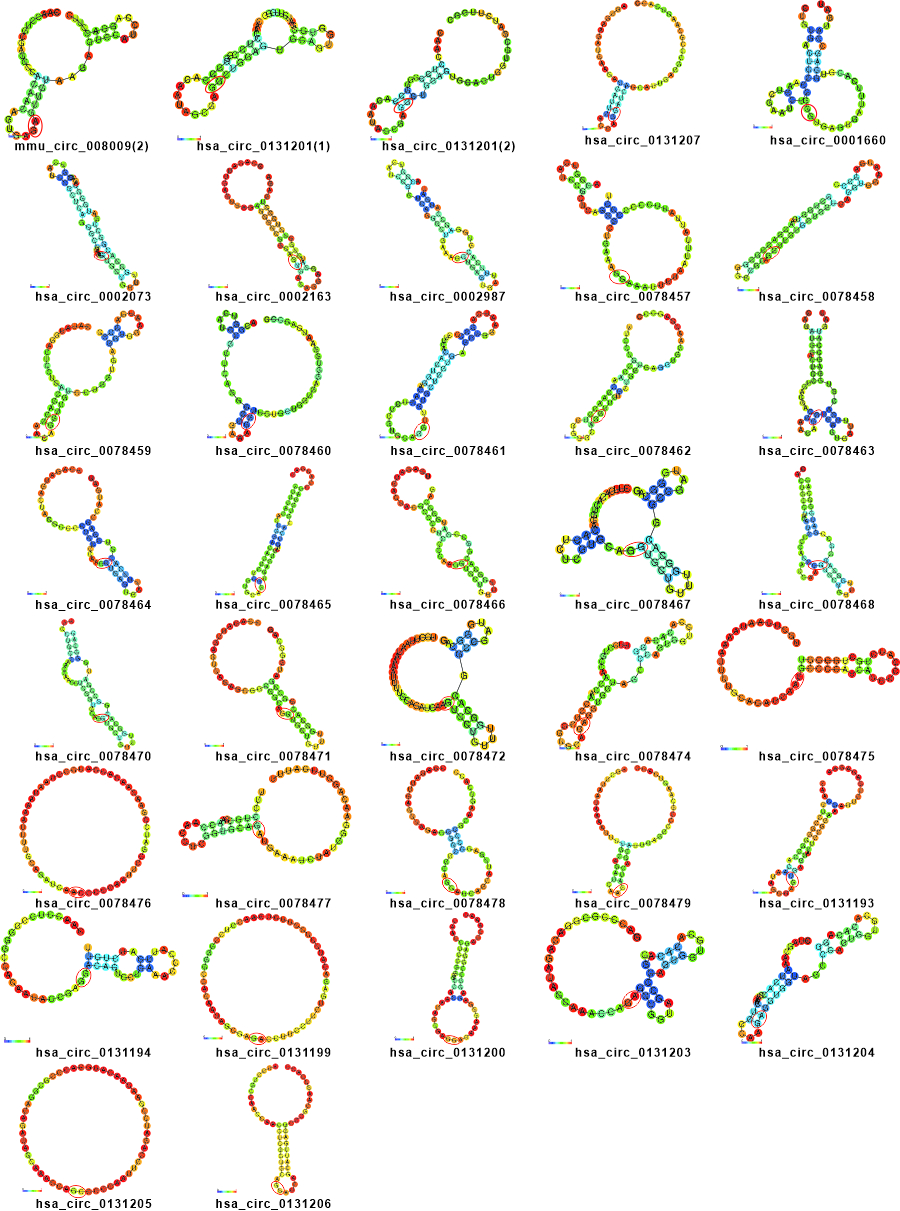


**Figure. S1. Computer mimic the difference in the second structure of circRNAs from parent *Tulp4*.**

**Figure. S2**


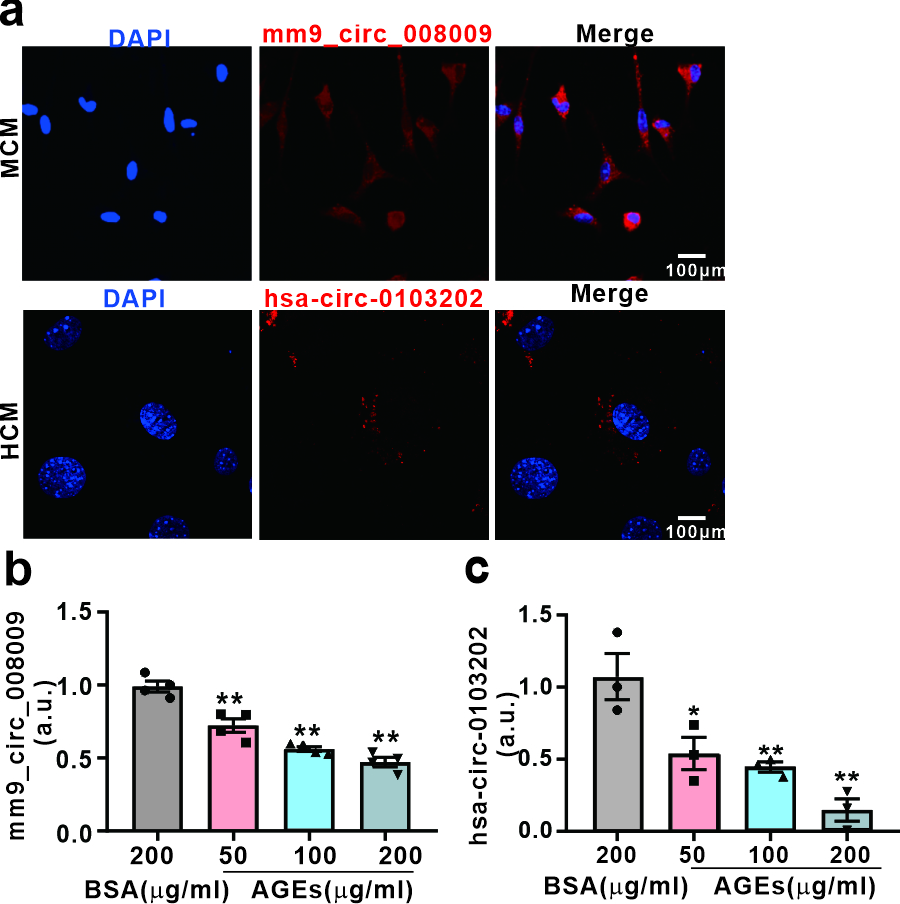


**Figure. S2. The effect of AGEs on the mm9_circ_008009 and has_circ_0131202 expression.**

1. The FISH assay was performed to detect the location of mm9_circ_008009 and has_circ_0131202 in the mouse and human heart cells, respectively.

(b) AGEs downregulated the mm9_circ_008009 expression in a dose-dependent. *^**^p ＜0.01* vs. BSA, n = 3.

(c) AGEs downregulated the has_circ_0131202 expression in a dose-dependent manner. *^*^p ＜ 0.05*, *^**^p ＜0.01* vs. BSA, n = 3.

**Figure. S3.**


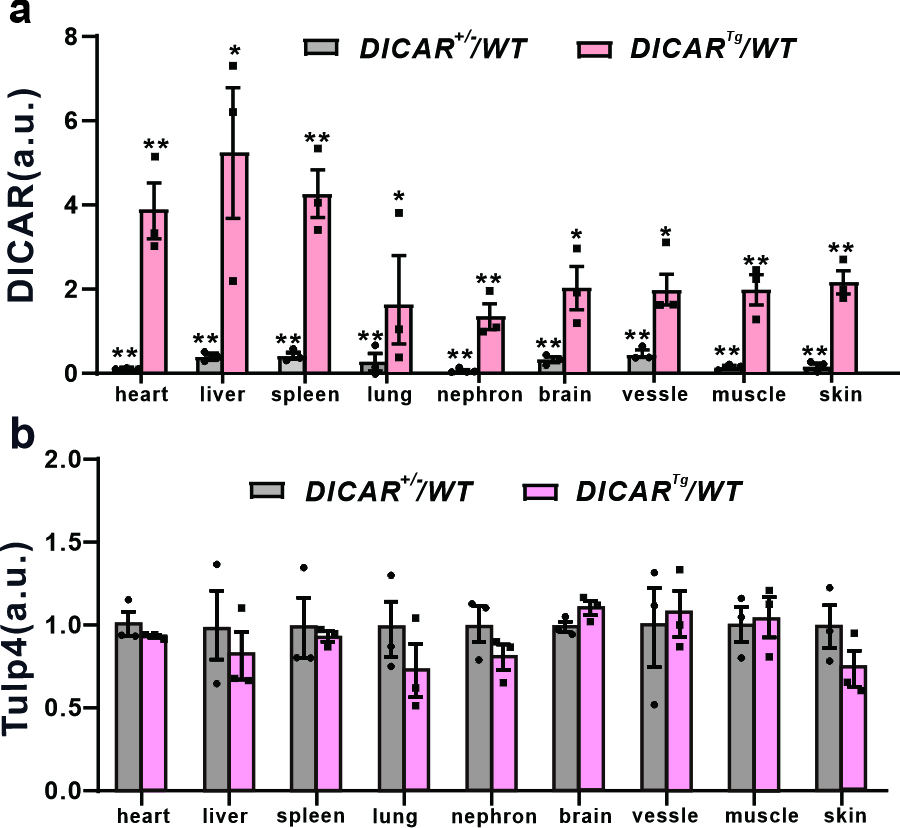


**Figure. S3. Identification of successfully founded *DICAR^+/-^* and *DICAR^Tg^* mouse model.**

1. qPCR detected DICAR expression in different tissues. *^*^P ＜ 0.05,* *^**^P ＜ 0.01* vs. WT, n = 3.
2. qPCR detected Tulp4 mRNA expression in different tissues. n = 3

**Figure. S4.**


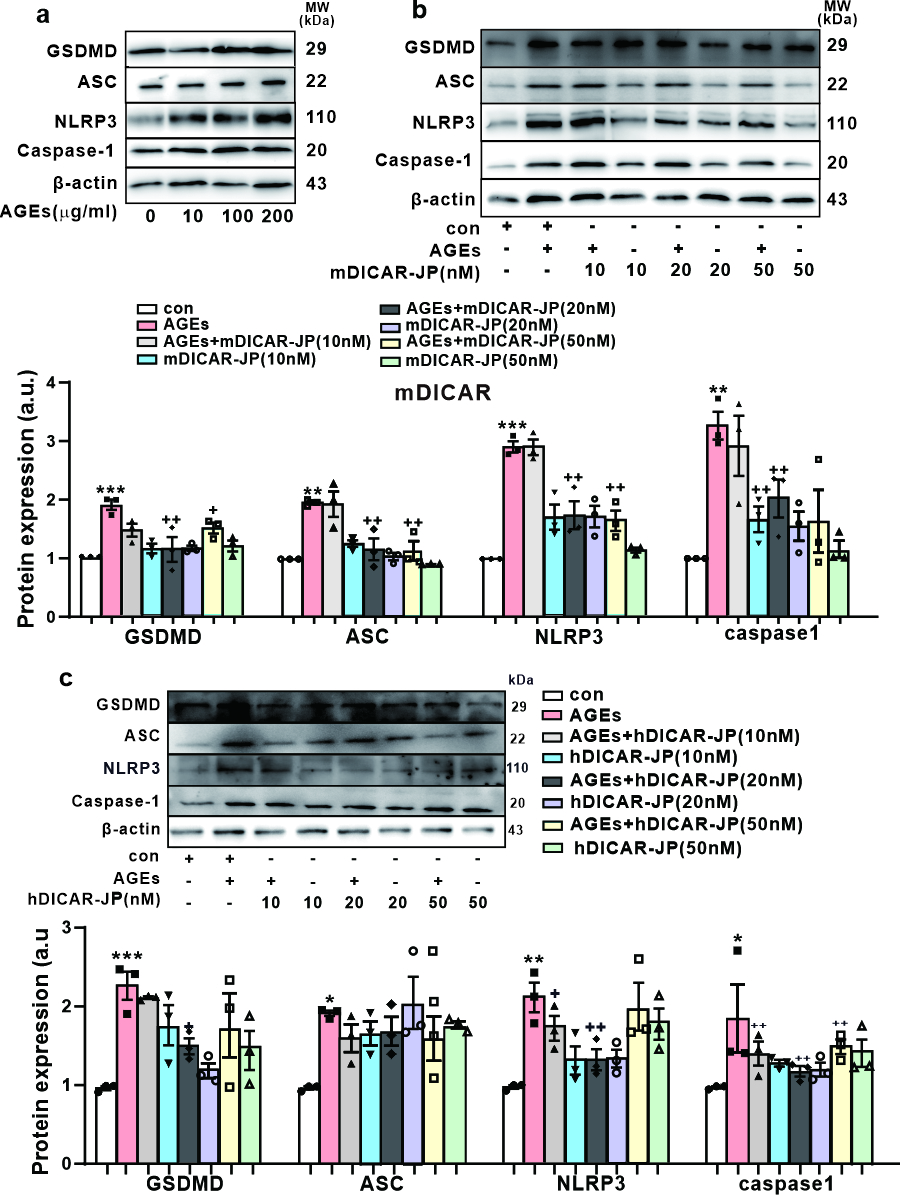


**Figure. S4. The effect of DICAR on the inhibition of pyroptosis of cardiomyocytes induced by AGEs.**

1. AGEs-induced HL-1 pyroptosis in a dose-dependent manner. n = 3–4.
2. mDICAR inhibited the reversal of the effect of AGEs on the HL-1 in a dose-dependent manner. *^*^P ＜ 0.05*, *^**^P ＜ 0.01*, *^***^P ＜ 0.001* vs. con; *^+^P ＜ 0.05*, *^++^P ＜ 0.01* vs. AGEs (200 µg/mL, 24 h); n = 3–4.
3. hDICAR inhibited the reversal of the effect of AGEs on the AC16 in a dose-dependent manner. *^*^P ＜ 0.05*, *^**^P ＜ 0.01*, *^***^P ＜ 0.001* vs. con; *^+^P ＜ 0.05*, *^++^P ＜ 0.01* vs. AGEs (200 µg/mL, 24 h); n = 3–4.

**Figure. S5.**


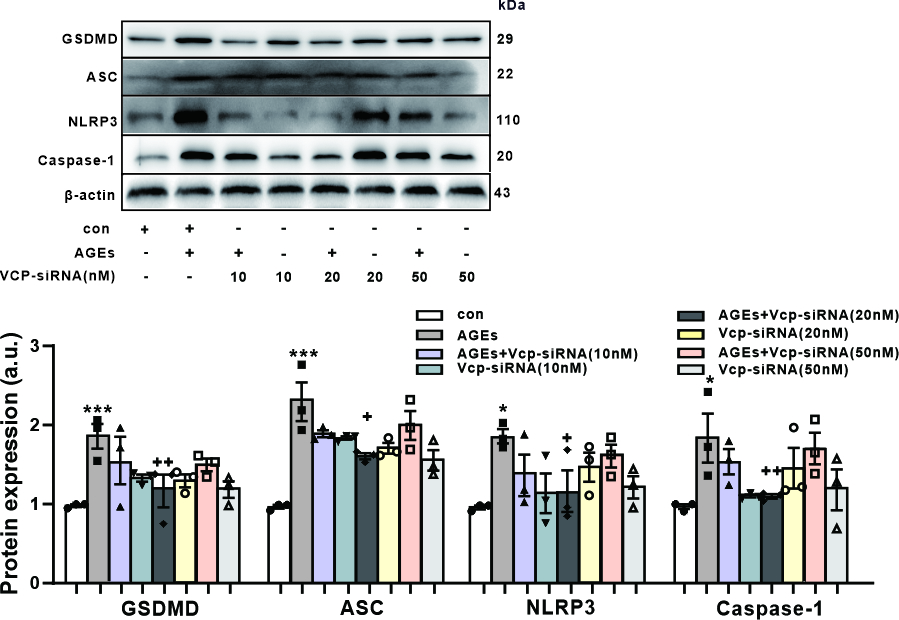


**Figure. S5. The inhibition of the effect of VCP-siRNA on the pyroptosis of HL-1 induced by AGEs.**

The pyroptosis was detected by ASC, caspase-1, GSDMD, and NLRP3 protein expression detected by WB, *^*^p ＜ 0.05*, *^***^p ＜ 0.001* vs. con; *^+^P＜ 0.05*, *^++^P ＜ 0.01* vs. AGEs (200 µg/mL, 24 h); n = 3–4.

**Figure. S6.**


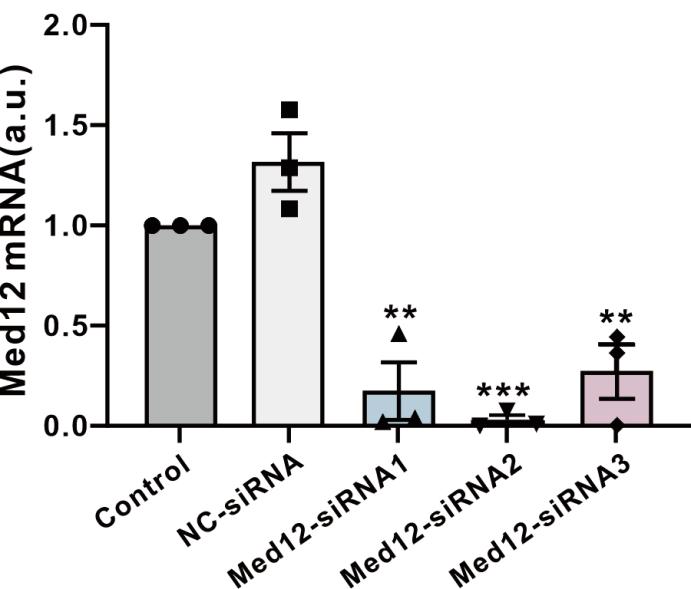


**Figure. S6. Med12 expression interrupted cell model founded.**

The interrupted effects of Med12-siRNA on Med12 expression detected by qPCR. *^**^P ＜ 0.01*, *^***^P ＜ 0.001* vs. NC-siRNA; n = 3–4.

**Supplementary Tables**

**Table S1. The detection of the DICAR-specific binding proteins by CHIRP-MS**

| Protein | db/db heart tissue | | WT heart tissue | | DICAR binding protein Fold Change |
| --- | --- | --- | --- | --- | --- |
|  | Tulp4 binding protein | DICAR binding  protein | Tulp4 binding protein | DICAR binding protein | db/db/WT |
| AIFM | 0 | 152138 | 0 | 3259094 | 0.046681 |
| 3HIDH | 0 | 0 | 0 | 553547 | 0 |
| PGBM | 0 | 0 | 0 | 877851 | 0 |
| NACA | 0 | 163030 | 0 | 1662008 | 0.098092 |
| VCP | 184773 | 287155 | 0 | 910187 | 0.31549 |
| MOES | 0 | 486971 | 0 | 1856162 | 0.262354 |
| IF4A1 | 309885 | 1241298 | 0 | 2728306 | 0.45497 |
| KAD2 | 0 | 120706 | 0 | 2173149 | 0.055544 |
| B2RXT3 | 0 | 177804 | 195714 | 2903948 | 0.061228 |
| ENPL | 0 | 1104477 | 0 | 9191303 | 0.120165 |
| TRI72 | 0 | 0 | 0 | 1764371 | 0 |
| ATPA | 13205434 | 1.39E+08 | 57529722 | 6.75E+08 | 0.205994 |
| ATPB | 7256879 | 73935412 | 31430421 | 2.27E+08 | 0.325275 |
| QCR8 | 96385628 | 18786930 | 112003 | 75282338 | 0.249553 |
| MYH6 | 964396 | 2679425 | 0 | 10338236 | 0.259176 |
| KCRB | 0 | 0 | 0 | 890856 | 0 |
| KCRS | 3521229 | 18299080 | 11951728 | 2.36E+08 | 0.077508 |
| MYL3 | 677315 | 65532772 | 0 | 3.12E+08 | 0.210362 |
| ADT1 | 11398077 | 5876799 | 6346335 | 76808958 | 0.076512 |
| Q8CGP4 | 8545191 | 36392631 | 0 | 30418303 | 1.196406 |
| TBA1C | 11346525 | 9541334 | 2626952 | 13177746 | 0.724049 |
| VIME | 23107304 | 20584724 | 0 | 9902824 | 2.078672 |
| NDUA4 | 3712477 | 10303468 | 11228266 | 51962445 | 0.198287 |
| TKT | 21952 | 30056 | 0 | 24206 | 1.241676 |
| D3Z0I3 | 2131183 | 2818265 | 1377943 | 11519331 | 0.244655 |
| VDAC1 | 0 | 1350666 | 0 | 0 | INF |
| Q91VB8 | 2556672 | 2930678 | 0 | 40377118 | 0.072583 |
| MYG | 926602 | 6053612 | 0 | 48634626 | 0.124471 |
| PLAK | 7313802 | 3993794 | 0 | 3981318 | 1.003134 |
| K1C14 | 3.97E+08 | 1.98E+08 | 41125644 | 29600755 | 6.684681 |
| E9Q9J0 | 3316360 | 6449274 | 0 | 14485101 | 0.445235 |
| ANXA5 | 0 | 0 | 105078 | 271963 | 0 |
| ACADL | 769620 | 9897253 | 5196006 | 69817344 | 0.141759 |
| PPIA | 0 | 1521979 | 0 | 4151735 | 0.366589 |
| LAMC3 | 2834921 | 2966795 | 387648 | 1322251 | 2.243746 |
| SPR2K | 1476124 | 0 | 0 | 0 | INF |
| ANXA2 | 79304 | 24132 | 24261 | 18089 | 1.33407 |
| DESP | 4731177 | 0 | 0 | 0 | INF |
| DPYL2 | 0 | 0 | 0 | 397417 | 0 |
| Q8C2Q7 | 0 | 253668 | 0 | 99783 | 2.542197 |
| Q8CBB6 | 2076379 | 2958268 | 1817511 | 2044638 | 1.446842 |
| BIP | 0 | 0 | 0 | 1207417 | 0 |
| FABP7 | 37205210 | 6802407 | 1.12E+08 | 23320355 | 0.291694 |
| H4 | 454434 | 675816 | 0 | 1065757 | 0.634118 |
| PRDX1 | 1574398 | 2356542 | 0 | 7790795 | 0.302478 |
| COX41 | 1395259 | 20717047 | 127709 | 1.04E+08 | 0.200018 |

**Table S2. The combined portion of DICAR-VCP contains three parts**

| DICAR | VCP | Binding model |
| --- | --- | --- |
| **Part** A |  |  |
| C1 | L12 | hydrogen bond |
| A2 | T14 | hydrogen bond |
| A3 | K18 | salt bridge |
| C4 | K20 | salt bridge |
| C4 | N21 | hydrogen bond |
| **Part B** |  |  |
| C43、C42 | K651、D484、D483、R487、Q479、K486 | Amino acid residue side chain network |
| **Part C** |  |  |
| G30 | R662、K663 | hydrogen bond |
| T39 | Q473 | Hydrophobicity |
|  | K164、R453、K389  N401、Q382 | hydrogen bond |
| A17 | E397 | hydrogen bond |
| A18 | N401 | static electricity |
| C19 | N401 | static electricity |
| A20 | E420 | hydrogen bond |
| A20 | K614 | salt bridge |
| G21 | E402 | hydrogen bond |
| T22 | D393、E402、R453 | Hydrophobicity |
| G23 | A446、S444 | hydrogen bond |
| A24 | D393、D393 | static electricity |
| G25, A26 | Q382 | static electricity |

**Table S3. Detection of different protein expressions in the heart tissues by LC-MS + PRM**

| Proteins | Positions within proteins | Localization prob | Score | Mass error [ppm] | Average *DICAR^+/-^* | Average WT | *DICAR^+/-^*/WT | P |
| --- | --- | --- | --- | --- | --- | --- | --- | --- |
| Myom1 | 1171 | 0.997 | 131.06 | -2.1191 | 3348166.67 | 9590867 | 0.35 | 0.037 |
| Mlrv | 111 | 0.974 | 76.679 | -2.3009 | 1154280 | 3453700 | 0.33 | 0.008 |
| Cavn2 | 102 | 1 | 88.009 | -0.1738 | 4570966.67 | 15033000 | 0.30 | 0.017 |
| Med12 | 1869 | 1 | 57.177 | -0.5763 | 188983333 | 786123333 | 0.24 | 0.013 |
| Myh6 | 1533 | 1 | 127.56 | 0.3736 | 3907836.67 | 20011667 | 0.20 | 0.013 |

**Table S4. The list of primer sequences used in the study**

| Gene name | Sequence |
| --- | --- |
| mm9_circ_008009 | Forward: 5'-TACTATGAAGAGGGATGGTTGG-3'  Reverse: 5’-AAATGGCACTTGATATGTTTGTT-3' |
| mm9_circ_006404 | Forward: 5'-ATTTCGGGCCATTCAGCGTC-3'  Reverse: 5'-GCAACACCACTCGGTTCTGC-3' |
| mm9_circ_013422 | Forward: 5'-TGGTGGATCCTGTTCGCCAG-3'  Reverse: 5'-AGACCAAGACTTGTGAGGCCA-3' |
| mm9_circ_009681 | Forward: 5'-TCTGAACGGCGAGATCCTGC-3'  Reverse: 5'-TGCGTTCCTGTTCTGGAAGCT-3' |
| mm9_circ_000903 | Forward: 5'-GGCCCCTCTGTTTACTGCTGA-3'  Reverse: 5'-GGCAGTGGCACGTTCTTTCT-3' |
| mouse Tulp4 | Forward: 5'-TCCTGCATGAATCTGATGGCA-3'  Reverse: 5'-GGGGAGGAGAATAATCATCCGA-3' |
| hsa_circ_0131202 | Forward:5'-CCAAGAGTGAGAAGGAGAAGCC-3'  Reverse: 5’-CCTGGAGTCTTACAAATCTCGCTA-3' |
| mouse GAPDH | Forward: 5'-CACTGAGCAAGAGAGGCCCTAT-3'  Reverse: 5'-GCAGCGAACTTTATTGATGGTATT-3' |
| human GAPDH | Forward: 5'-GACAGTCAGCCGCATCTTCT-3'  Reverse: 5’-GCGCCCAATACGACCAAATC-3' |
| CHIRP-MS probe |  |
| P1 | ACTCTTACAACTCTCACTGTTGTG |
| P2 | aggagccatcagattattca |
| P3 | gcttctatgtgcagaaaagg |
| P4 | caaccccaacagtggaaagg |
| P5 | actggagctattctttaggg |
| P6 | aggaatctgttcctttcaga |
| P7 | actgacagttccaattagta |
| P8 | taccaaagatgtcttcctgt |
| P9 | attaattagtctccgtctgc |
| P10 | aatggaccaagtagtttcct |
