# Supplementary material for: CircRNA DICAR as a novel endogenous regulator for diabetic cardiomyopathy and diabetic pyroptosis of cardiomyocytes
Source: Signal Transduct Target Ther. 2023 Mar 8;8:99. doi: 10.1038/s41392-022-01306-2 (PMC9992392; doi:10.1038/s41392-022-01306-2)
Supplement: Supplementary file 2 — orignial wb picutre [file 41392_2022_1306_MOESM2_ESM.pptx]

## Slide 1
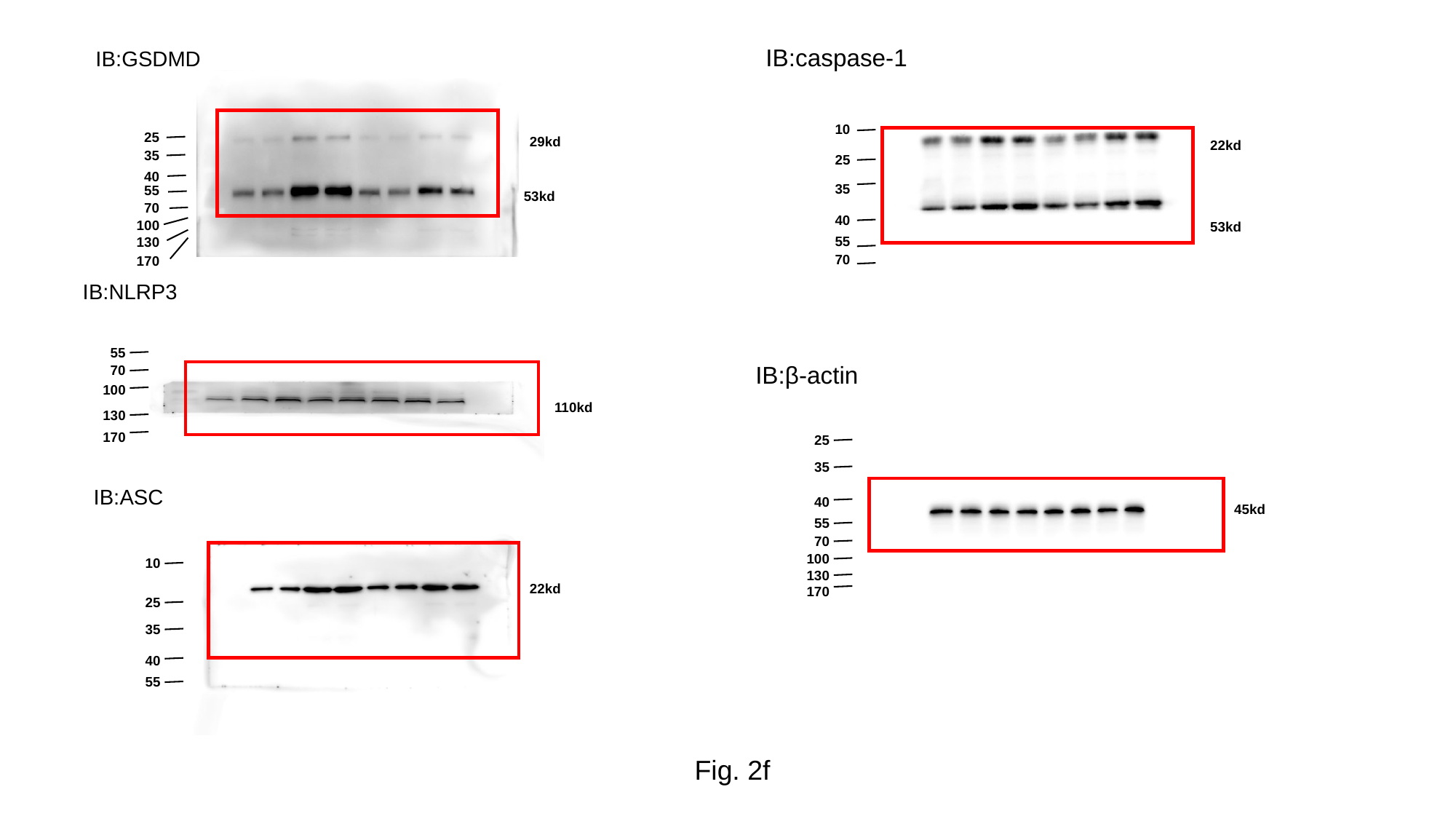

IB:caspase-1
IB:GSDMD
10
25
29kd
22kd
35
25
40
35
55
53kd
70
40
100
53kd
55
130
70
170
IB:NLRP3
55
IB:β-actin
70
100
110kd
130
170
25
35
IB:ASC
40
45kd
55
70
100
10
130
22kd
170
25
35
40
55
Fig. 2f

## Slide 2
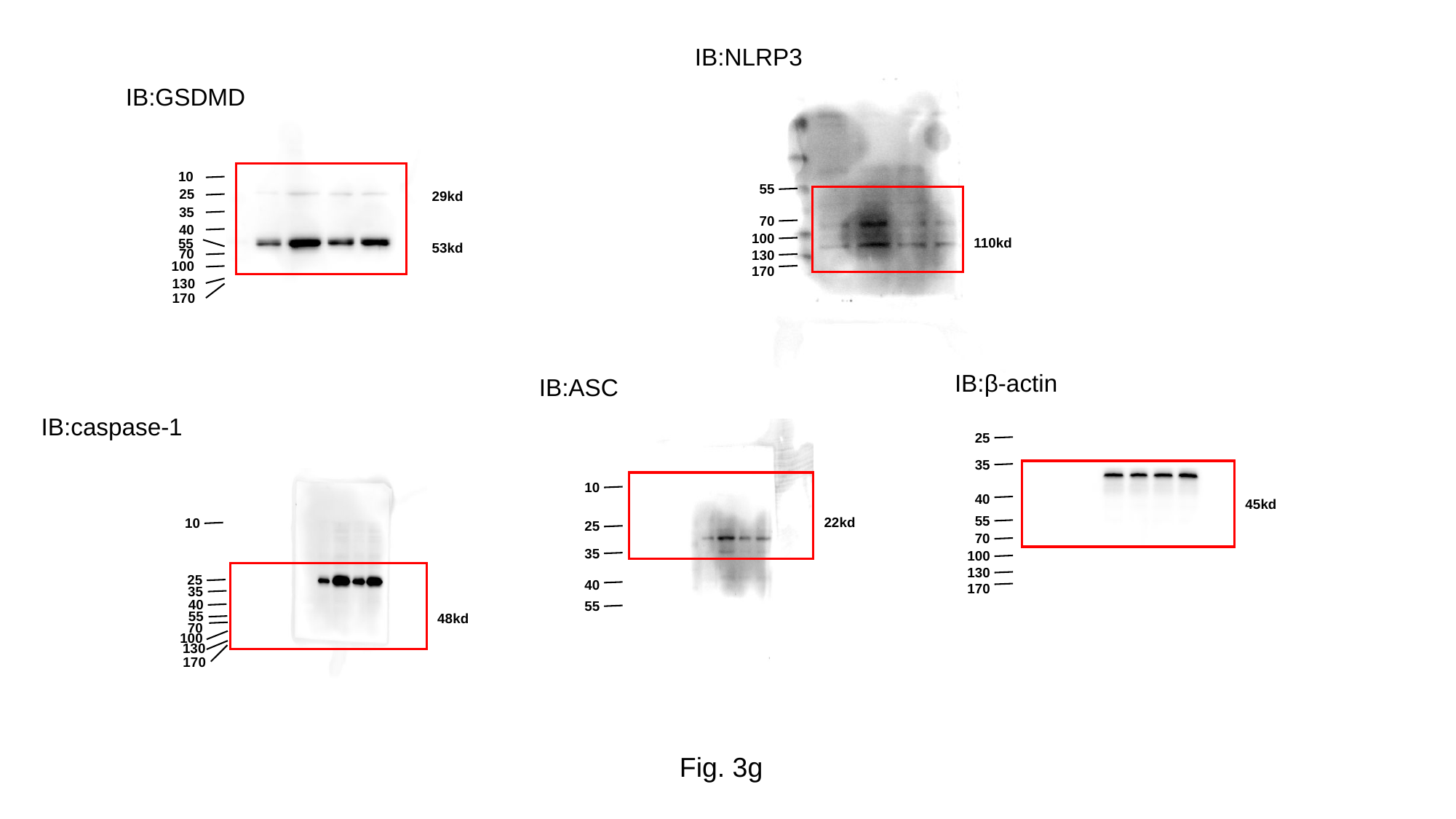

IB:NLRP3
IB:GSDMD
10
55
25
29kd
35
55
70
40
70
100
110kd
55
110kd
53kd
100
70
130
100
130
170
170
130
170
IB:β-actin
IB:ASC
IB:caspase-1
25
35
10
40
45kd
55
22kd
10
25
70
35
100
130
25
40
170
35
40
55
55
48kd
70
100
130
170
Fig. 3g

## Slide 3
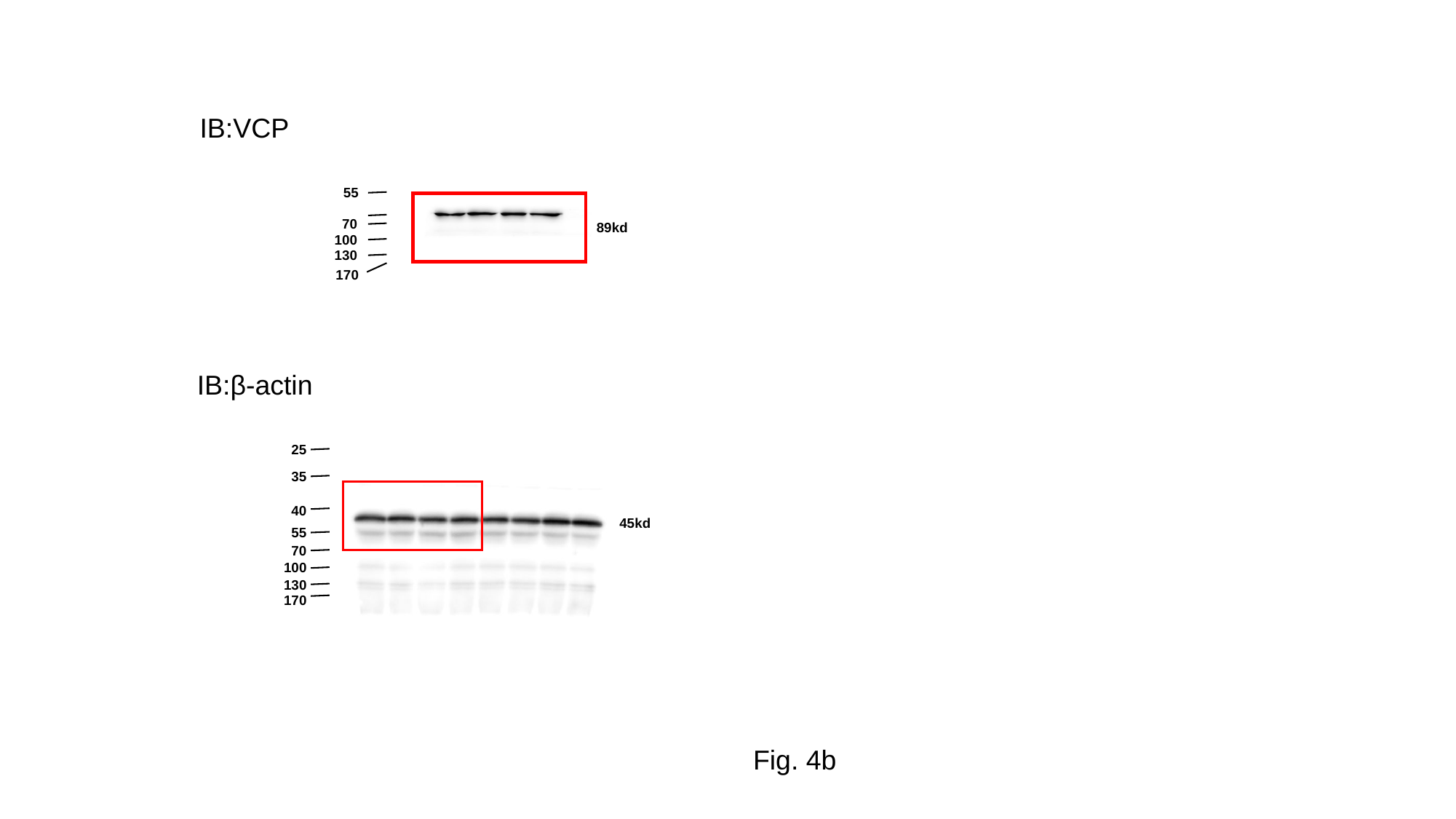

IB:VCP
55
70
89kd
100
130
170
IB:β-actin
25
35
40
45kd
55
70
100
130
170
Fig. 4b

## Slide 4
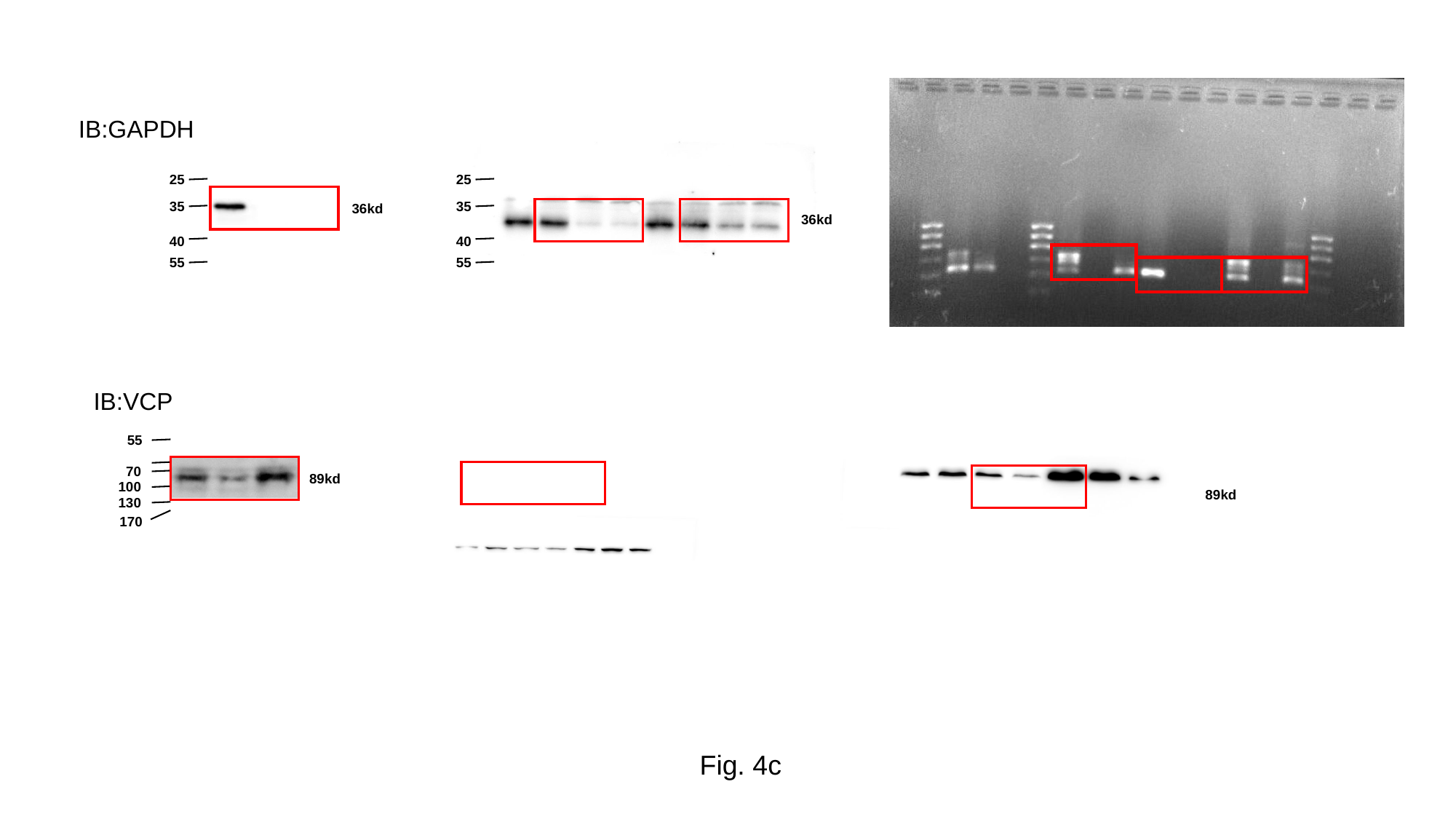

IB:GAPDH
25
25
35
35
36kd
36kd
40
40
55
55
IB:VCP
55
70
89kd
100
89kd
130
170
Fig. 4c

## Slide 5
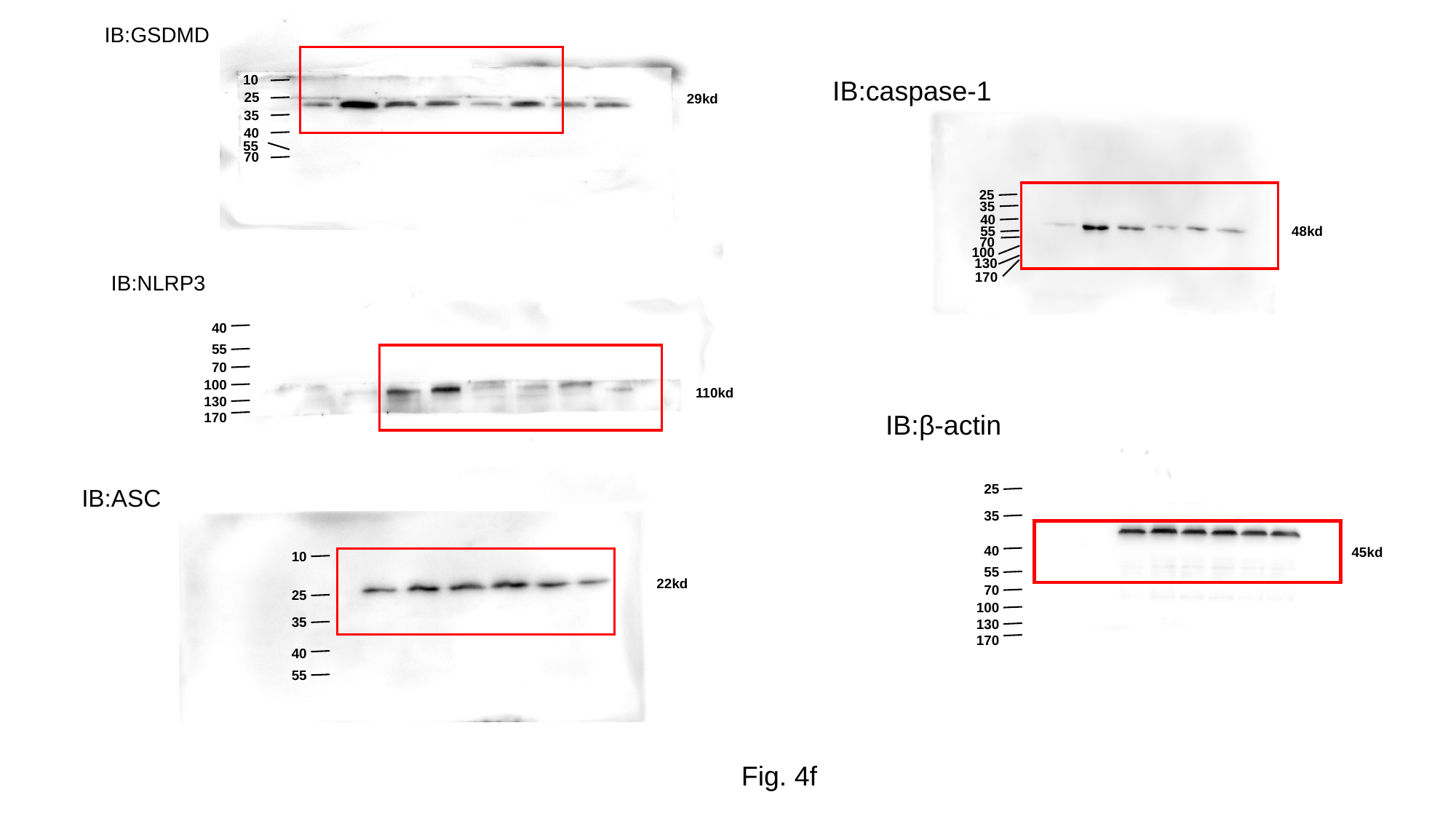

IB:GSDMD
10
IB:caspase-1
25
29kd
35
40
55
70
25
35
40
55
48kd
70
100
130
170
IB:NLRP3
40
55
70
100
110kd
IB:β-actin
130
170
25
IB:ASC
35
40
45kd
10
55
22kd
70
25
100
35
130
170
40
55
Fig. 4f

## Slide 6
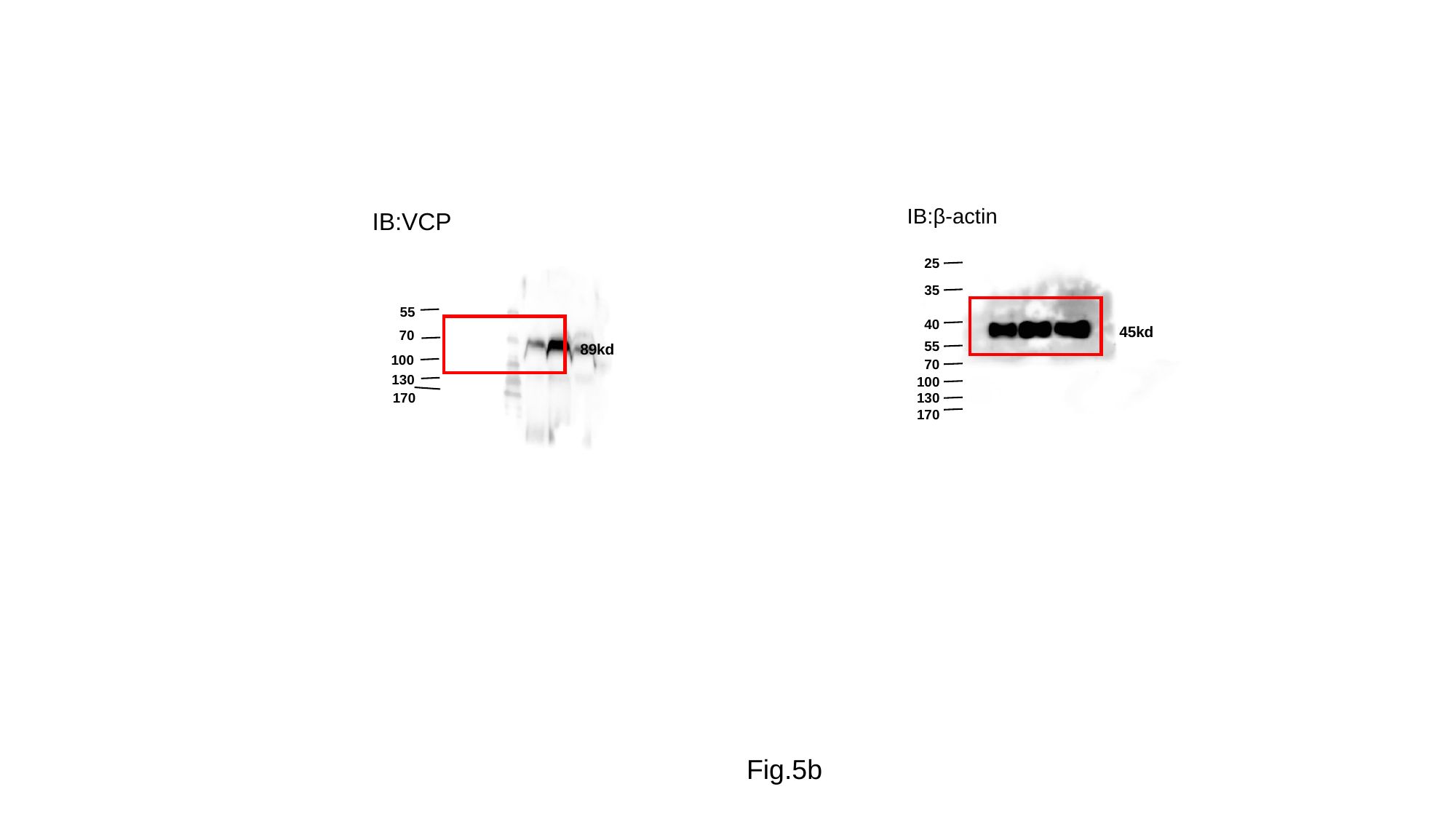

IB:β-actin
IB:VCP
25
35
55
40
45kd
70
55
89kd
100
70
130
100
170
130
170
Fig.5b

## Slide 7
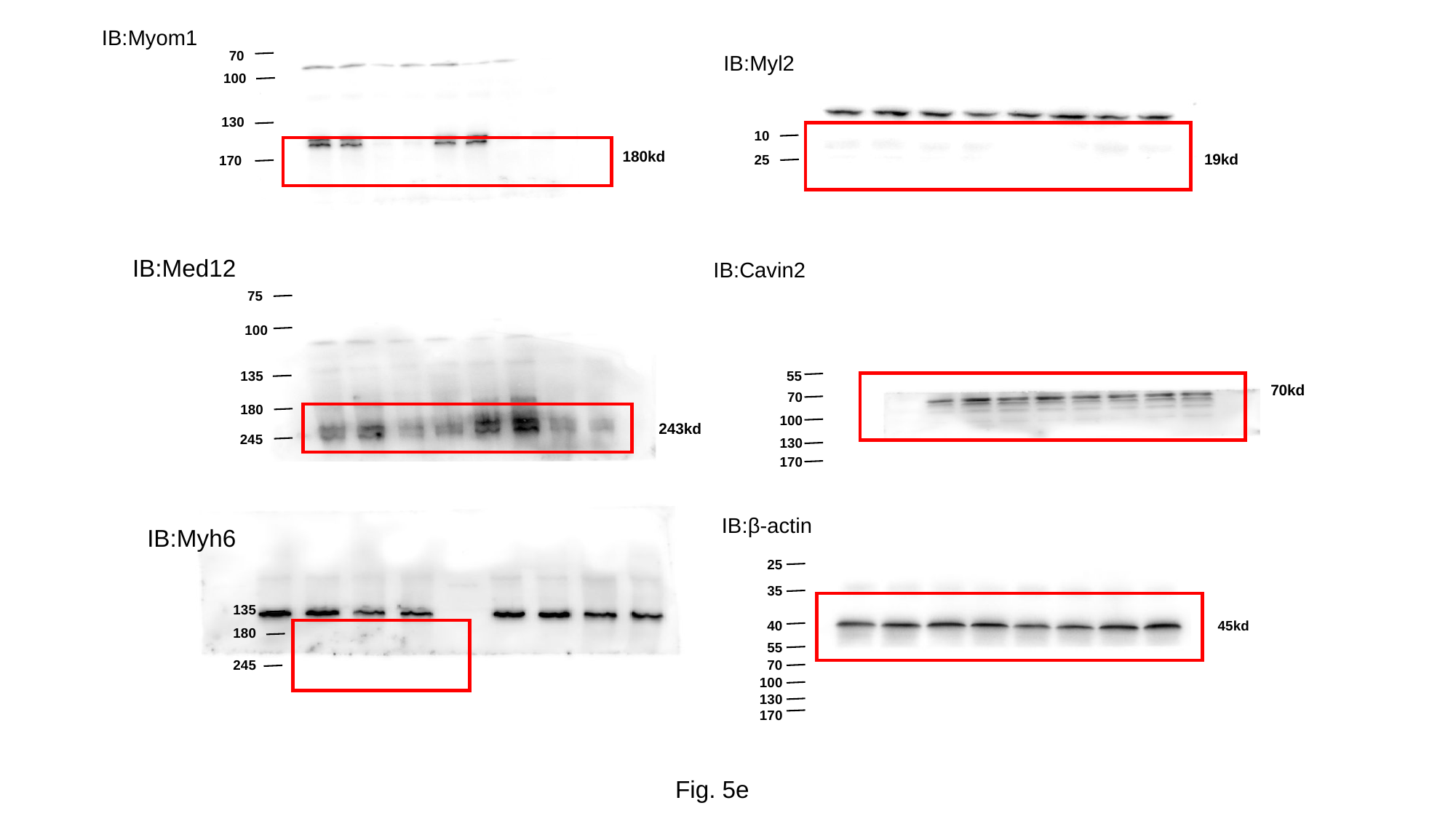

IB:Myl2
IB:Myom1
70
100
130
10
180kd
19kd
25
170
IB:Med12
IB:Cavin2
75
100
135
55
70kd
70
180
100
243kd
245
130
IB:Myh6
170
IB:β-actin
25
35
135
40
45kd
180
55
224kd
245
70
100
130
170
Fig. 5e

## Slide 8
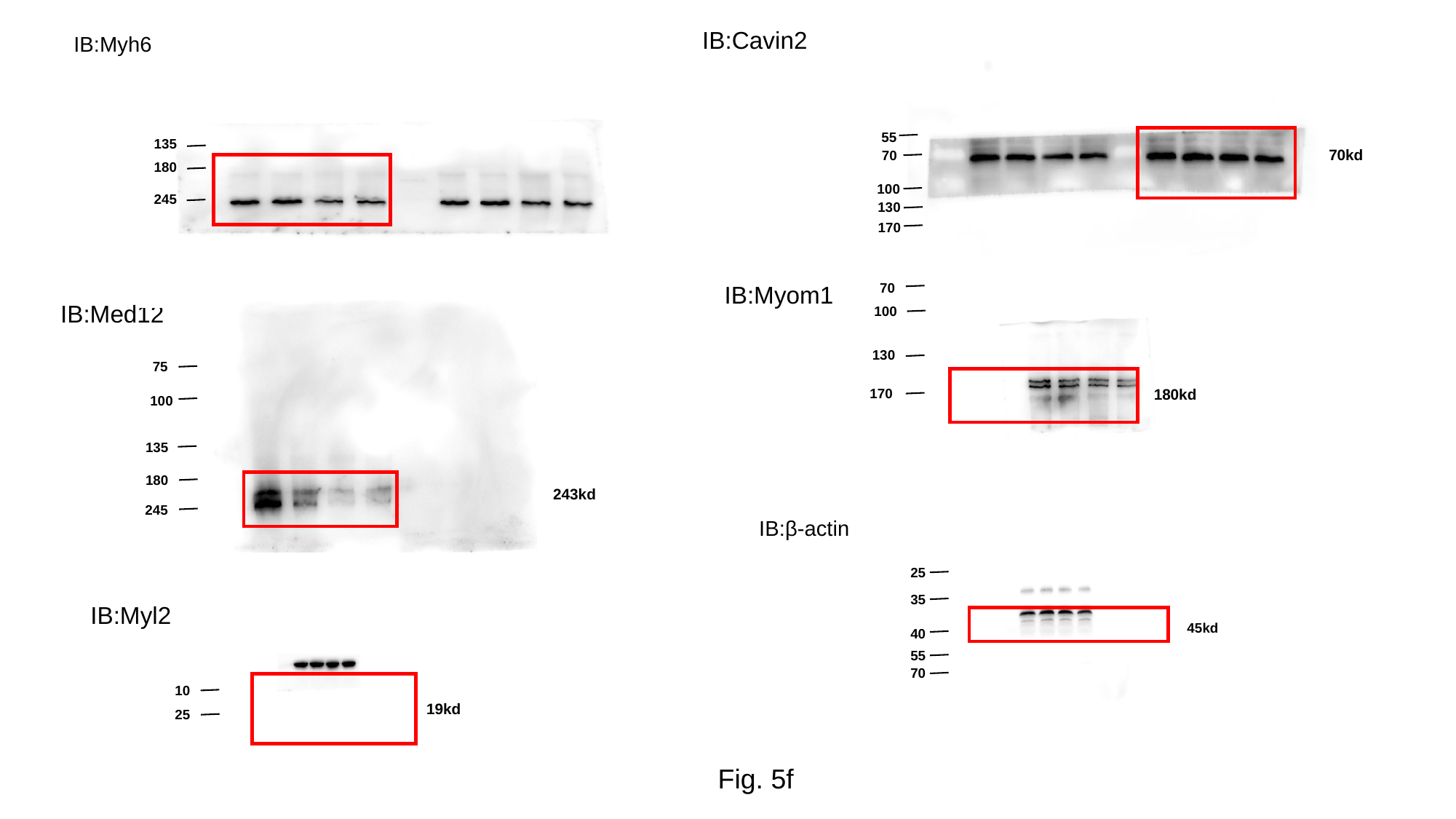

IB:Cavin2
IB:Myh6
55
135
70kd
70
180
224kd
100
245
130
170
IB:Myom1
70
IB:Med12
100
130
75
170
180kd
100
135
180
243kd
245
IB:β-actin
25
35
IB:Myl2
45kd
40
55
70
10
19kd
25
Fig. 5f

## Slide 9
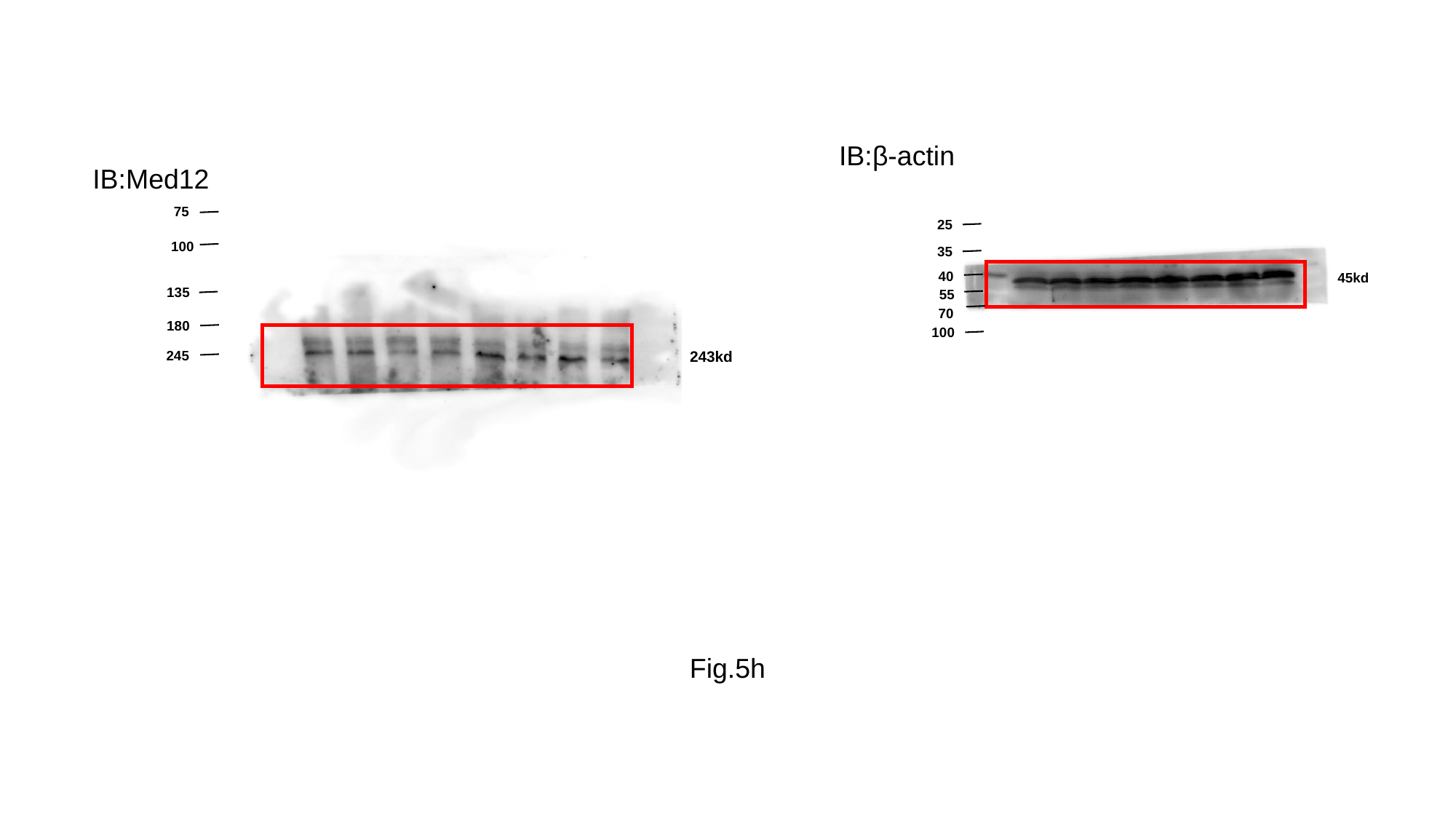

IB:β-actin
IB:Med12
75
25
100
35
40
45kd
135
55
70
180
100
243kd
245
243kd
Fig.5h

## Slide 10
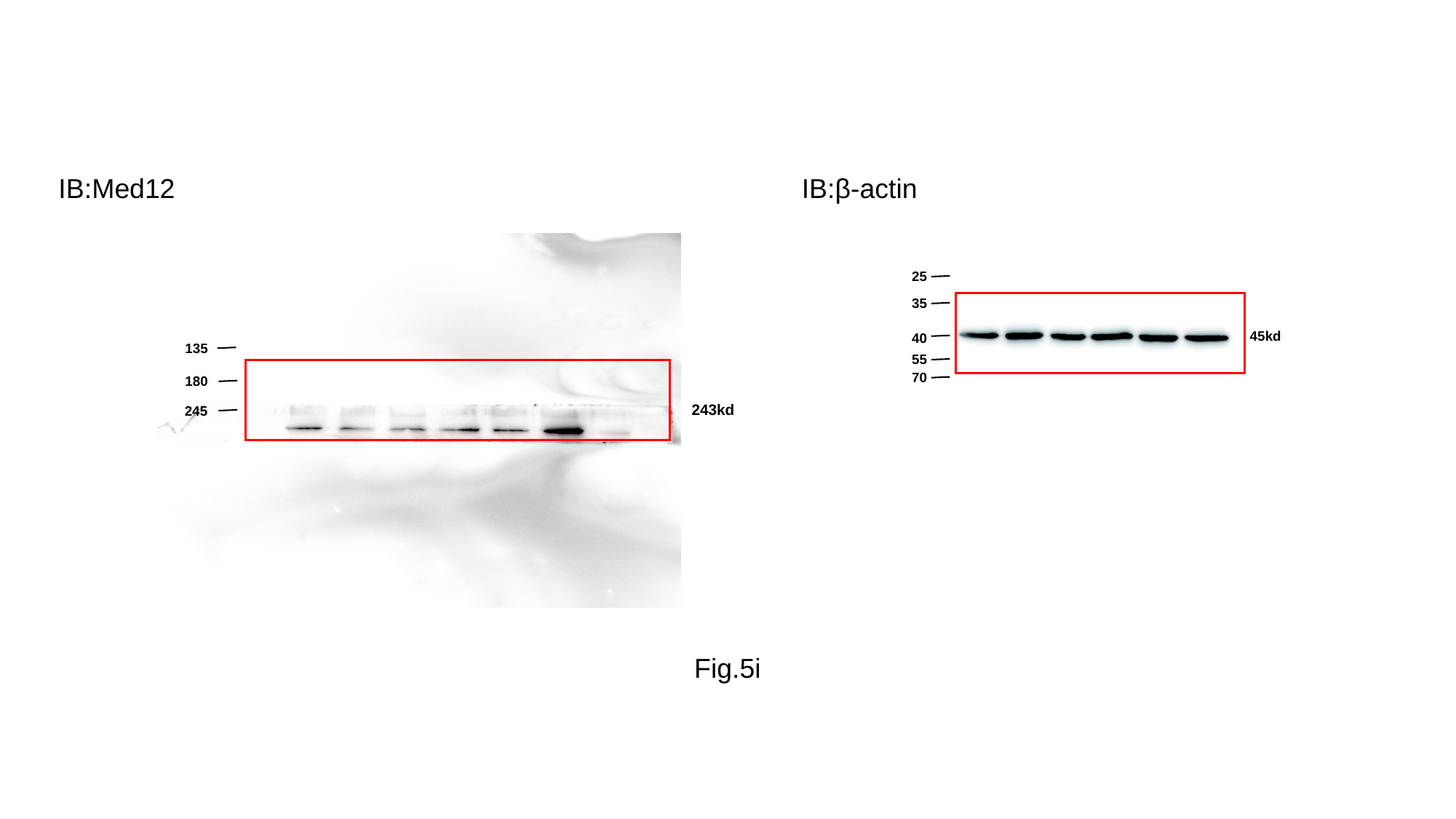

IB:Med12
IB:β-actin
25
35
45kd
40
135
55
70
180
243kd
245
Fig.5i

## Slide 11
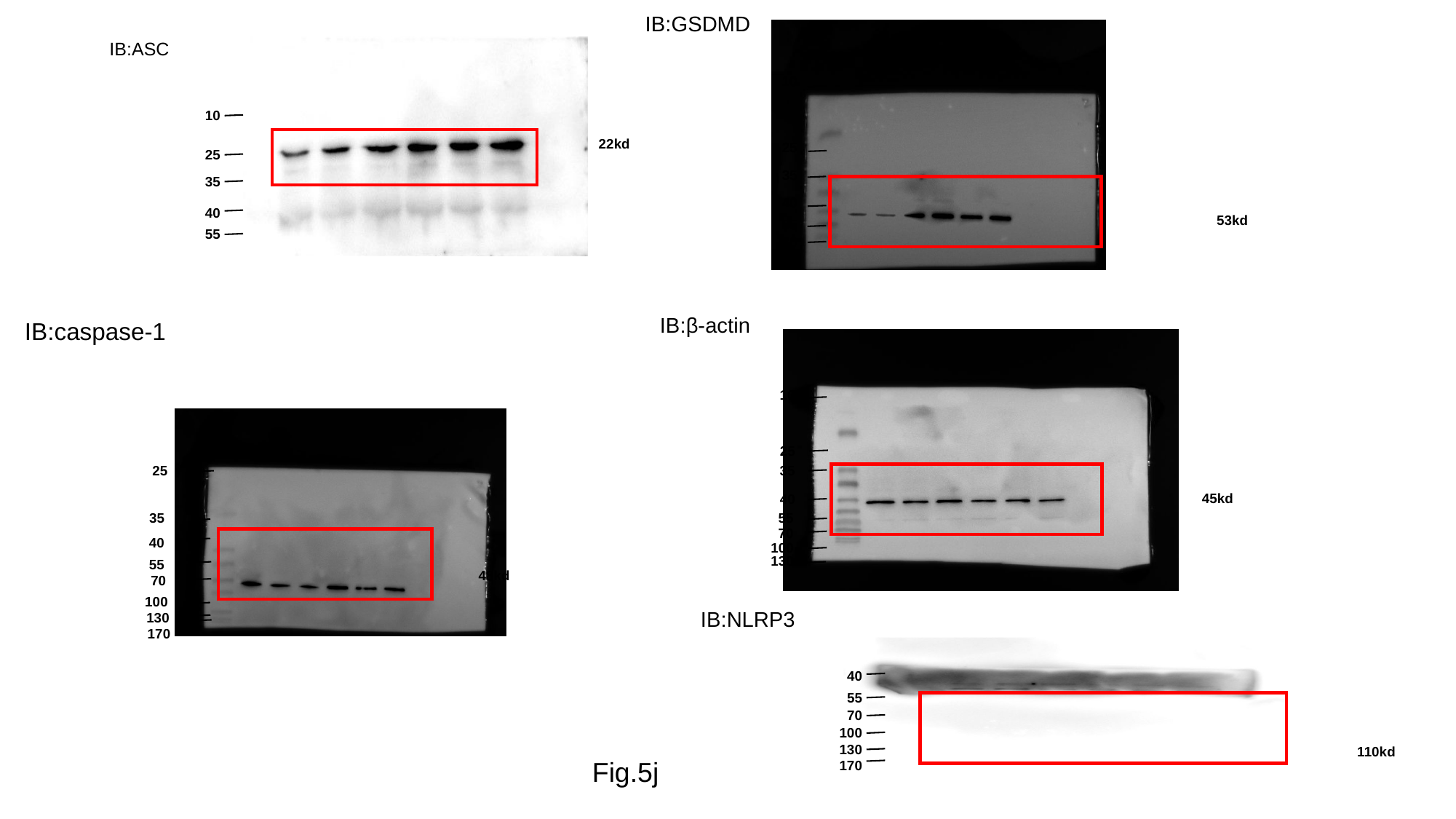

IB:GSDMD
IB:ASC
10
10
22kd
25
25
35
35
40
40
53kd
55
55
70
IB:β-actin
IB:caspase-1
10
25
25
35
45kd
40
35
55
70
40
100
130
55
48kd
70
100
IB:NLRP3
130
170
40
55
70
100
130
110kd
Fig.5j
170

## Slide 12
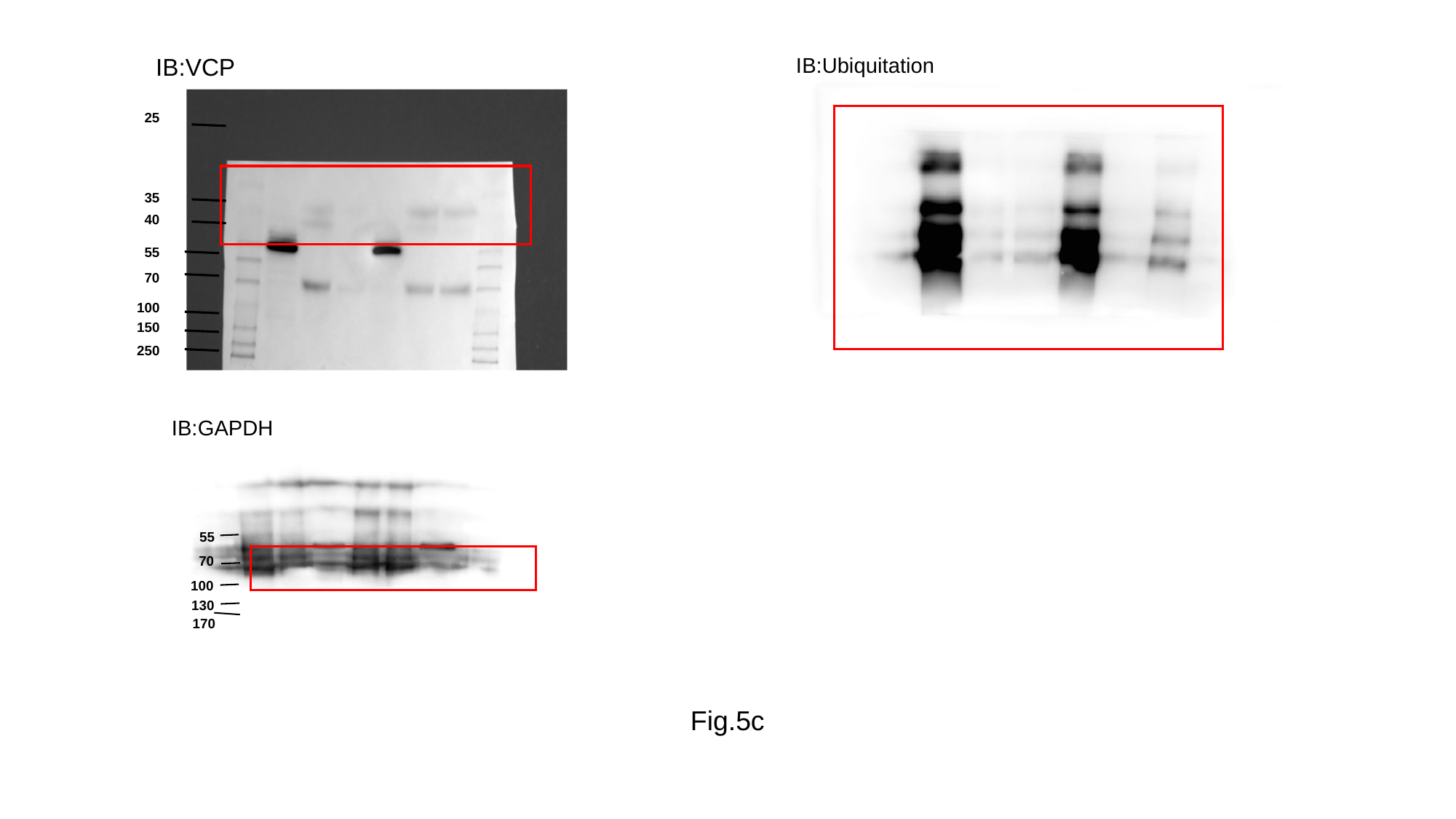

IB:VCP
IB:Ubiquitation
25
35
40
55
70
100
150
250
IB:GAPDH
55
70
100
130
170
Fig.5c

## Slide 13
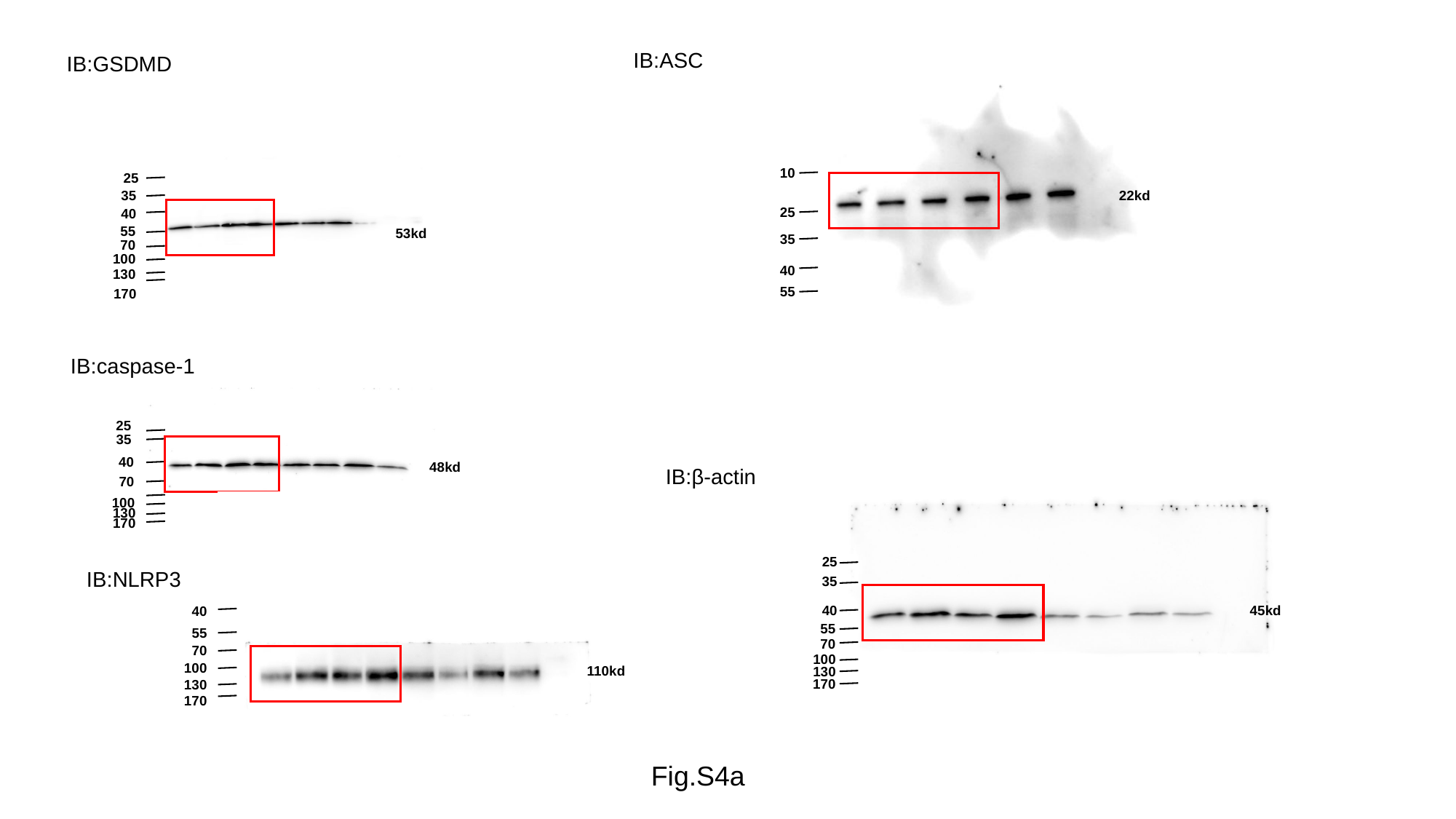

IB:ASC
IB:GSDMD
10
25
35
22kd
25
40
55
53kd
35
70
100
40
130
55
170
IB:caspase-1
25
35
40
48kd
IB:β-actin
70
100
130
170
25
IB:NLRP3
35
40
45kd
40
55
55
70
70
100
100
110kd
130
170
130
170
Fig.S4a

## Slide 14
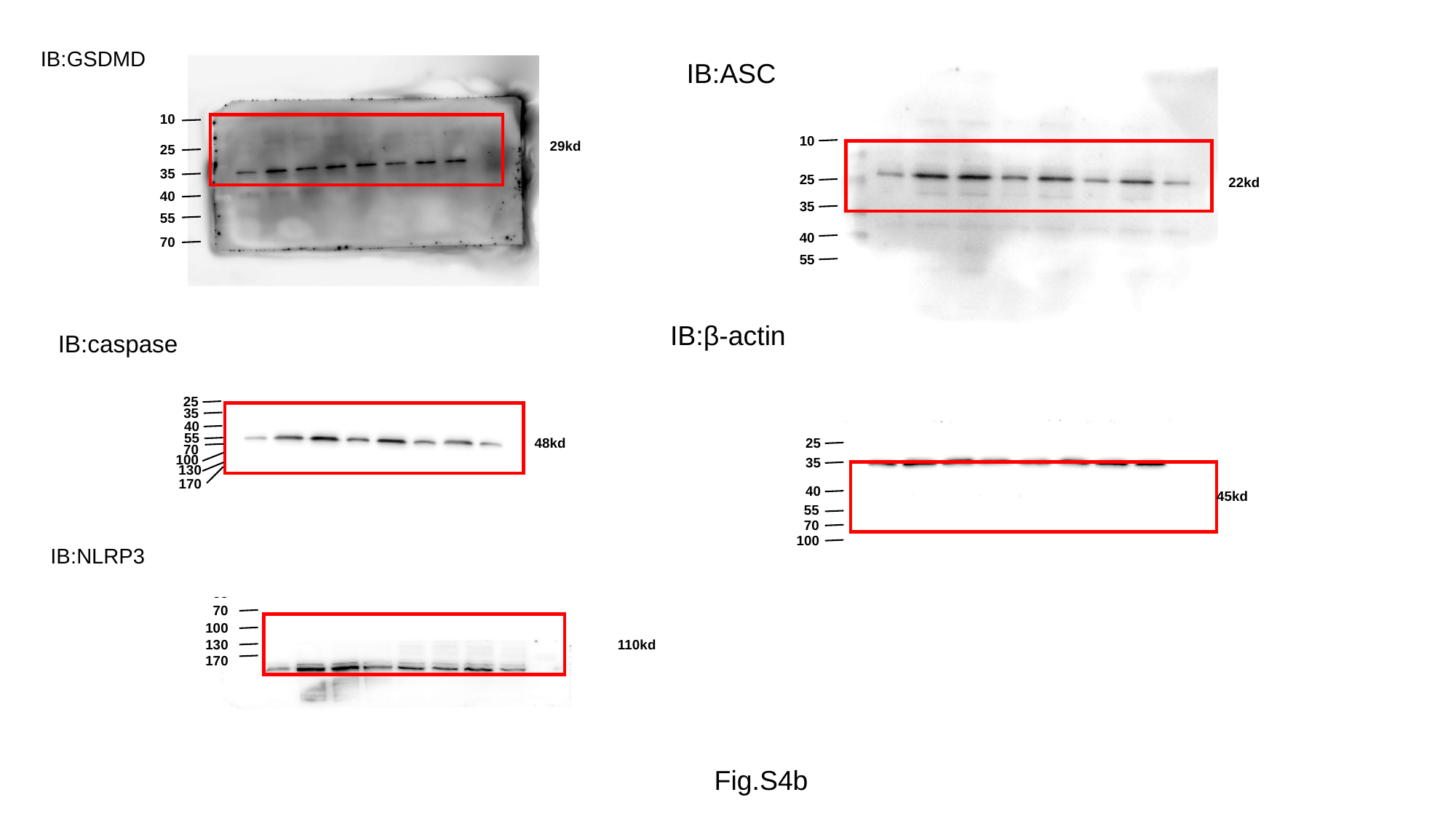

IB:GSDMD
IB:ASC
10
10
29kd
25
35
25
22kd
40
35
55
40
70
55
IB:β-actin
IB:caspase-1
25
35
40
55
48kd
25
70
100
35
130
170
40
45kd
55
70
100
IB:NLRP3
40
55
70
100
130
110kd
170
Fig.S4b

## Slide 15
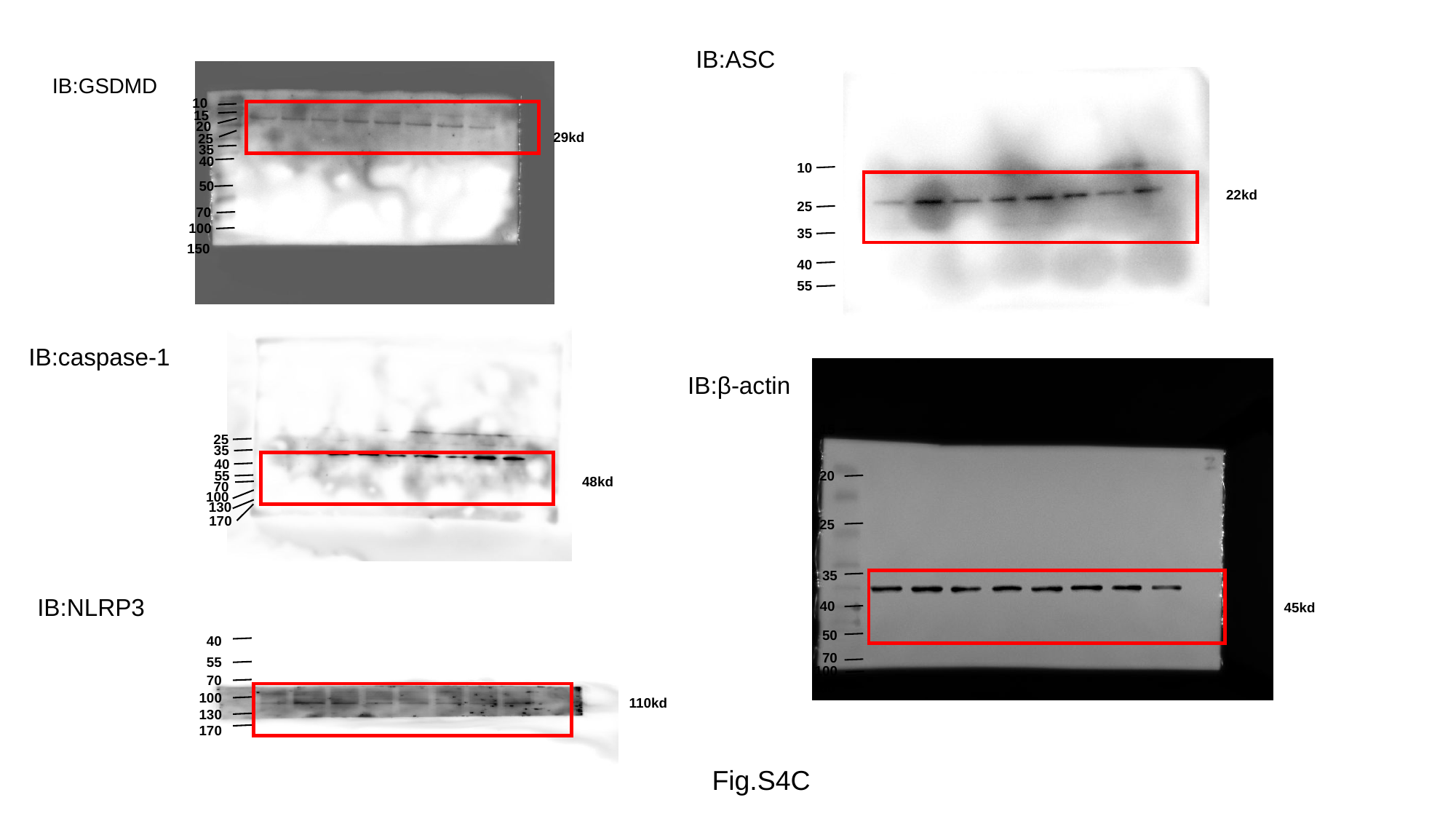

IB:ASC
IB:GSDMD
10
15
20
29kd
25
35
40
10
50
22kd
25
70
100
35
150
40
55
IB:caspase-1
IB:β-actin
15
25
35
40
55
20
48kd
70
100
130
170
25
35
IB:NLRP3
40
45kd
50
40
70
55
100
70
150
100
110kd
130
170
Fig.S4C

## Slide 16
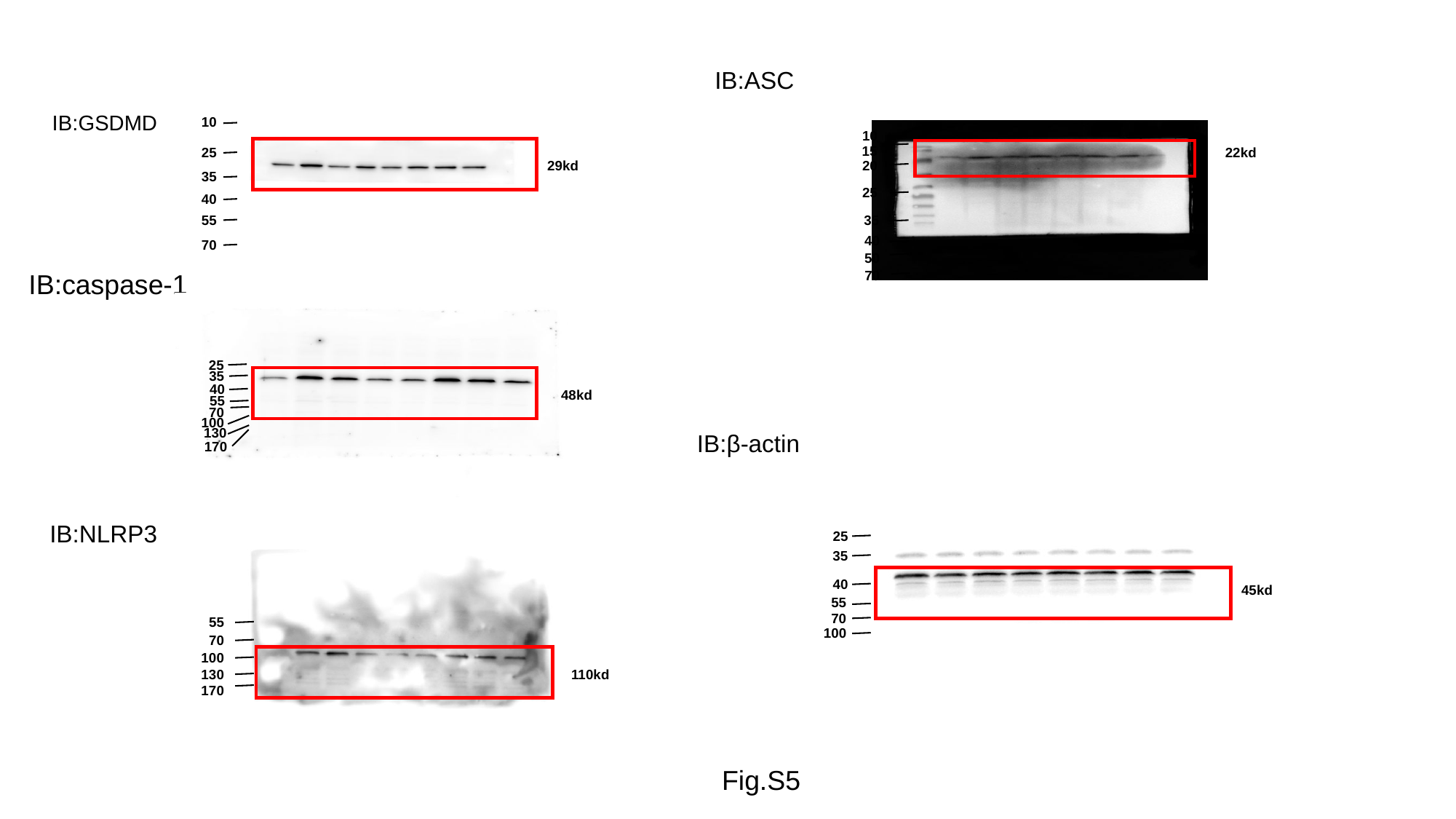

IB:ASC
IB:GSDMD
10
10
15
25
22kd
29kd
20
35
25
40
35
55
40
70
50
IB:caspase-1
70
25
35
25
40
35
55
40
70
48kd
55
100
70
130
100
170
130
IB:β-actin
170
IB:NLRP3
25
35
40
45kd
55
70
55
100
70
100
130
110kd
170
Fig.S5
